# Mfsd2a is important for maintaining epidermal homeostasis

**DOI:** 10.1073/pnas.2531159123

**Published:** 2026-02-19

**Authors:** Bernice H. Wong, Kunal Mishra, Cheen Fei Chin, Dwight L. A. Galam, Bryan C. Tan, Mei Ding, Federico Torta, Jacques Behmoaras, Alvin W. C. Chua, David L. Silver

**Affiliations:** ^a^Signature Research Program in Cardiovascular and Metabolic Disorders, Duke–National University of Singapore Medical School, Singapore 169857, Singapore; ^b^Centre for Computational Biology, Duke–National University of Singapore Medical School, Singapore 169857, Singapore; ^c^Singapore Lipidomics Incubator, Life Sciences Institute, Singapore 117456, Singapore; ^d^Precision Medicine Translational Research Programme and Department of Biochemistry, Yong Loo Lin School of Medicine, National University of Singapore, Singapore 117597, Singapore; ^e^Department of Plastic, Reconstructive and Aesthetic Surgery, Singapore General Hospital, Singapore 169856, Singapore; ^f^Musculoskeletal Sciences Academic Clinical Programme, Duke–National University of Singapore Medical School, Singapore 169857, Singapore

**Keywords:** transporters, phospholipid, fatty acids, skin, metabolism

## Abstract

The skin epidermis is a multilayered lipid rich organ completely reliant on exogenous delivery and uptake of the essential fatty linoleate for the synthesis of acylceramides required for barrier function. Disruption of the epidermal skin barrier is a common clinical feature for prevalent diseases such as atopic dermatitis and psoriasis. The granulosum layer of the epidermis contains specialized secretory keratinocytes that synthesize and secrete lamellar bodies (LB), making it likely that this epidermal layer has a high demand for phosphatidylcholine. While the pathway for acylceramide biosynthesis and roles in barrier formation has been largely elucidated, the mechanisms by which keratinocytes acquire phospholipid for the demands of lipid barrier maintenance and repair are not entirely understood. Here, we demonstrate that Mfsd2a, a lysophosphatidylcholine (LPC) transporter, is predominantly expressed in keratinocytes and mediates the uptake of a plasma-derived LPC fluorescent probe. Epidermal-specific deficiency of Mfsd2a in mice resulted in dermatitis and defective desquamation of the epidermis, and inducible deletion of *Mfsd2a* in primary mouse keratinocytes in vitro prevented their epidermal stratification. Untargeted lipidomic analysis indicated that Mfsd2a deficiency resulted in a significantly altered phospholipidome, with specific reductions in linoleic acid in phosphatidylcholine and triglycerides in the epidermis. Functional studies using primary human keratinocytes demonstrated that LPC-oleate and LPC-linoleate promoted keratinocyte differentiation in a Mfsd2a-dependent manner. Our findings identify a pathway by which keratinocytes acquire plasma-derived LPC via Mfsd2a transport for the maintenance of normal keratinocyte phosphatidylcholine pools and for optimal keratinocyte differentiation.

## Significance

Disruption of the epidermal skin barrier is a common clinical feature for prevalent diseases such as atopic dermatitis and psoriasis. Epidermal function is reliant on dietary linoleic acid and has a high demand for phospholipids, but whether epidermal keratinocytes take up plasma-derived phospholipid such as the abundant plasma lysophosphatidylcholines (LPC) is not known. This study utilized mouse models of epidermal Mfsd2a deficiency, histological and gene expression analysis, untargeted lipidomics, and human keratinocyte cultures, demonstrating that the epidermal keratinocytes acquire plasma-derived LPC via Mfsd2a mediated transport; and this pathway is important for maintaining a normal phospholipidome and proper epidermal differentiation. The findings in this study raise the possibility that LPCs might have therapeutic benefit for epidermal health.

The skin consists of highly organized layers of cells that can be broadly classified into the epidermis, the underlying dermis, and subcutaneous fat. The epidermis is made up of four sublayers of keratinocytes that follow a vertical path of differentiation from the basal cell layer containing the stem cell compartment, into the spinous, granular, and lastly the outermost corneum layer (1). The turnover of normal human epidermis takes about 40 to 56 d and is eight times faster in psoriatic skin (2, 3). The mouse epidermis is replaced more rapidly with turnover times of 8 to 10 d, with the differentiation of granular cells into terminally differentiated corneocytes devoid of nuclei requiring just 5 d (4). This highly regulated process of corneocyte desquamation and replacement, coupled with the secretion of a specialized lipid lamellar matrix is essential for the maintenance of the epidermal skin barrier (1, 5). Disruption of the epidermal skin barrier is a common clinical feature for prevalent diseases such as atopic dermatitis, psoriasis, or lamella ichthyosis (6).

Formation of the epidermal lipid barrier is mediated by keratinocytes of the granular layer that synthesize and secrete lamellar bodies (LB) (7, 8). LB are lysosome-related organelles derived from the Golgi Apparatus that are rich in phospholipids, cholesterol, sphingomyelin, and glucosylceramides and acylceramides (8–11). The essentiality of acylceramides for barrier function is due to the esterification of linoleic acid to the omega-hydroxylated ultralong fatty acids on ceramides. The series of enzymes that mediate acylceramide synthesis has been elucidated. This biosynthetic pathway involves the uptake and storage of linoleic acid by keratinocytes in cytosolic triglyceride lipid droplets (LDs) through the action of DGAT2 (12). The hydroxylation of ultralong fatty acids of ceramides is mediated by CYP4F22 that involves the transfer of linoleic acid from triglyceride to the omega-hydroxylated ceramides as the final step. This step is mediated by the action of the transacylase Pnpla1 that is activated by ABHD5/CGI-58 (13, 14). Biallelic loss-of-function variants in CYP4F22 (mouse Cyp4f39), PNPLA1, and ABHD5 result in autosomal recessive congenital ichthyosis (13–16). Mouse deficiency models for DGAT2, Cyp4f39 (human CYP4F22), PNPLA1, and ABHD5 result in early postnatal lethality due to dehydration consequential to a nonfunctional epidermal barrier (13, 15, 16).

In addition to acylceramides, formation of a functional epidermal barrier requires the packaging of phospholipids and secretory phospholipase A_2_s (PLA_2_s), beta-glucocerebrosidase, and sphingomyelinase into LB at the granulosum layer. The lipid content of these LB are processed and secreted extracellularly to form the lamellar membrane where the phospholipid component is hydrolyzed by Pla2g2f (17, 18), ultimately resulting in a secreted lamellar layer poor in phospholipid (10, 19–22). Perturbations of the epidermal barrier, such as in acute removal of the corneum’s lipid by tape-stripping, or treatment with 10% SDS or acetone with gentle abrasion, coincides with an early response of increased epidermal fatty acid and cholesterol synthesis (23–25), with epidermal lipid synthesis returning to normal once the barrier is restored. This is consistent with observations that preformed LB are recruited to the corneum almost immediately after barrier disruption, followed by the synthesis and secretion of lamellar body contents to restore barrier integrity (26). This suggests that a high demand for phospholipid is required for lamellar body formation. Mechanisms by which keratinocytes acquire phospholipid for the demands of lipid barrier maintenance and repair are not entirely understood.

The lysolipid transporter Major Facilitator Superfamily Domain containing 2a (Mfsd2a), is a sodium-dependent lysolipid transporter that is highly expressed in the endothelium of the blood–brain and blood–retinal barriers (27, 28). Mfsd2a preferentially transports lysophosphatidylcholines (LPC) having mono- and polyunsaturated fatty acid moieties (27, 29). LPCs are produced by the liver and circulate in plasma bound to albumin (30). Mfsd2a is the primary transporter by which the brain and eye acquires the essential fatty acid docosahexaenoic acid (DHA) in the form of LPC-DHA, where gene targeted *Mfsd2a* deficiency models have significantly reduced DHA levels in brain and retina and present with severe microcephaly (27, 28, 31–33). Consistent with these findings, humans with loss-of-function variants in *MFSD2A* are characterized by severe microcephaly and hypomyelination and intellectual disability (a.k.a. Familial Microcephaly 15, Autosomal) (34–37). Mfsd2a expression is not confined to the brain or eye vasculature but expressed in oligodendrocyte precursor cells of the brain (38) and in tissues such as liver, intestine, lymphocytes, kidney, and lung (39–42). It is important to note that Mfsd2a is highly expressed in alveolar type 2 cells (AT2) of the mouse and human lung (41, 43–45). AT2 cells, like keratinocytes of the granulosum layer of the epidermis, produce LB full of phospholipid surfactant. We demonstrated that Mfsd2a is important in surfactant recycling by AT2 cell by mediating the reuptake of LPC generated by phospholipase activity of surfactant phospholipid in the alveolar space, and that this transport process is important for maintaining pulmonary surfactant levels (41). Here, we present evidence that Mfsd2a is expressed in differentiated suprabasal keratinocytes where it transports LPCs derived from plasma and is important for maintaining normal desquamation.

## Results

### Mfsd2a Is Predominately Expressed in Differentiated Keratinocytes of the Epidermis.

**Fig. 1.**
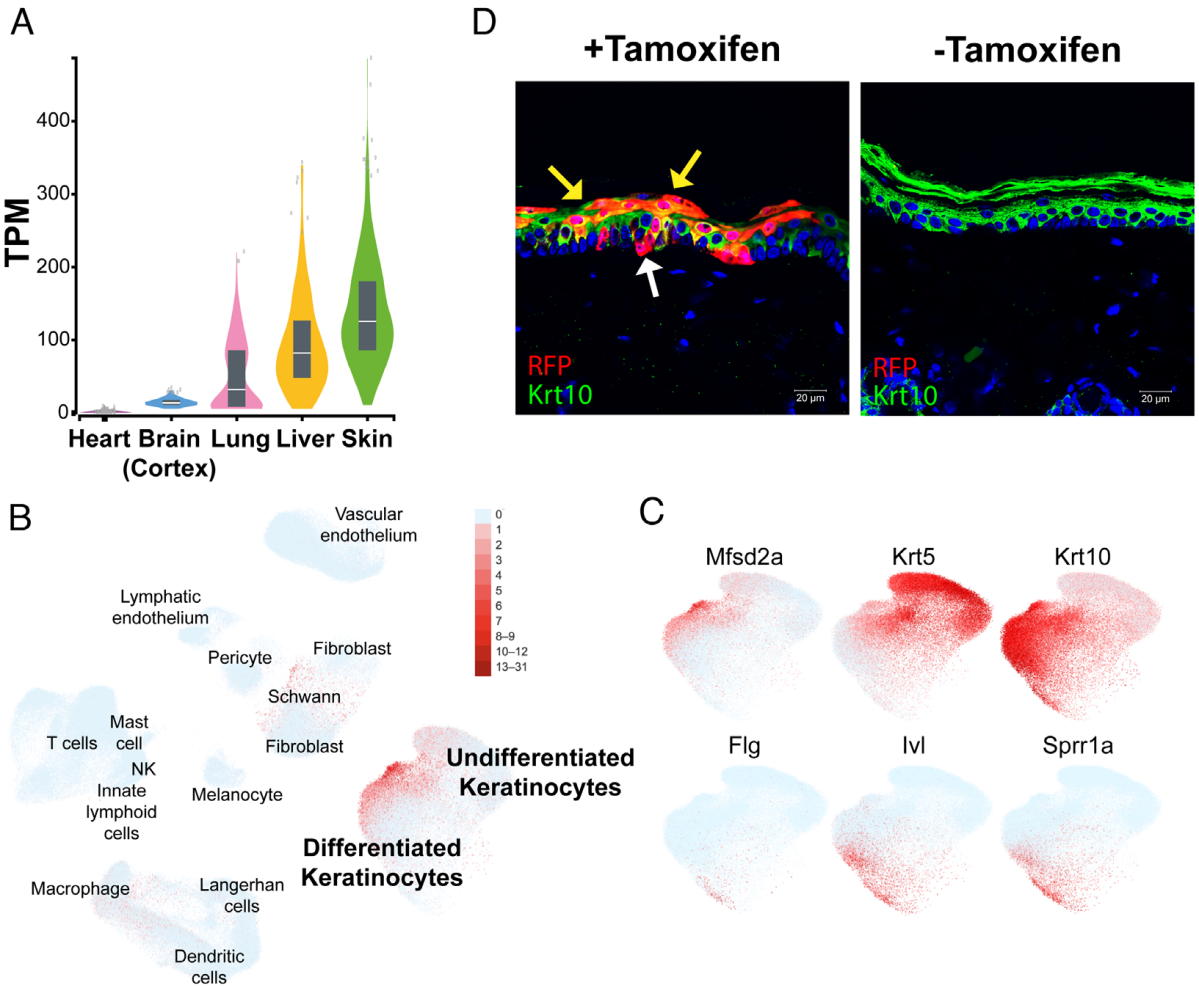
MFSD2A is expressed in human and mouse keratinocytes. (*A*) Violin plot showing *MFSD2A* expression in TPM (transcripts per million) in selected human tissues. Box plots are shown as median and 25th and 75th percentiles, where points are displayed as outliers if they are above or below 1.5 times the interquartile range. Image modified from GTex Portal, Broad Institute. (*B*) UMAP visualization of human skin scRNA-seq data from Reynolds et al. (48). *MFSD2A* expressing cells are shown in red. (*C*) *MFSD2A* and various skin differentiation markers from the same dataset (48) shown in red. Krt5 (basal keratinocytes), Krt10 (suprabasal keratinocytes), Flg, Ivl, Sprr1a (granular keratinocytes). (*D*) A tamoxifen-inducible lineage tracing mouse line for Mfsd2a (Mfsd2a^ERT2cre^TdTomato). RFP (red) indicates Mfsd2a expression in basal (white arrow) and differentiated keratinocytes (yellow arrows) in mice injected with tamoxifen (+Tmx). Krt10 (green) identifies suprabasal keratinocytes. Skin of untreated mice (−Tmx) serves as a negative control. *n* = 3 per treatment group. (Scale bar, 20 µm.)

Through data-mining of publicly available RNA-seq databases, we found that the human skin expresses the highest level of *MFSD2A* when compared against the brain, lung, and liver (Fig. 1*A*, GTex Portal, Broad Institute). Single-cell RNA-seq (scRNA-seq) databases indicated that *Mfsd2a* is expressed in suprabasal and differentiated keratinocytes of the interfollicular epidermis (IFE) of mice (46, 47) and humans (Fig. 1*B* and *SI Appendix*, Fig. S1) (48, 49). *Mfsd2a* expression was increased with keratinocyte differentiation in mice, with the highest expression found in the granular cell layer, the last living layer of the IFE (46, 47). In humans, *MFSD2A* is enriched in differentiated keratinocytes (Fig. 1*C* and *SI Appendix*, Fig. S1 *C* and *H*) as indicated with its coexpression with differentiation markers Keratin-1 (KRT1) and Keratin-10 (KRT10) (Fig. 1*C* and *SI Appendix*, Fig. S1 *B* and *G*). Interestingly, examination of scRNA-seq studies of human skin revealed that *MFSD2A* expression was decreased in keratinocytes of eczema and psoriatic skin (*SI Appendix*, Fig. S1) (48, 49), raising the possibility that MFSD2A is functionally important for epidermal health. To confirm some of these scRNA-seq findings, we utilized a Mfsd2a lineage tracing mouse line designated as Mfsd2a^ERT2cre^ that was crossed to the cre-inducible TdTomato reporter line (42, 50). In line with scRNA-seq datasets, the Mfsd2a^ERT2cre^TdTomato reporter was found to be predominantly expressed in differentiated keratinocytes with rare labeling of basal cells (Fig. 1*D*) of adult epidermis as well as keratinocytes prenatally at e18.5 (*SI Appendix*, Fig. S2).

### Inducible Postnatal Mfsd2a Deficiency Leads to Defective Desquamation.

**Fig. 2.**
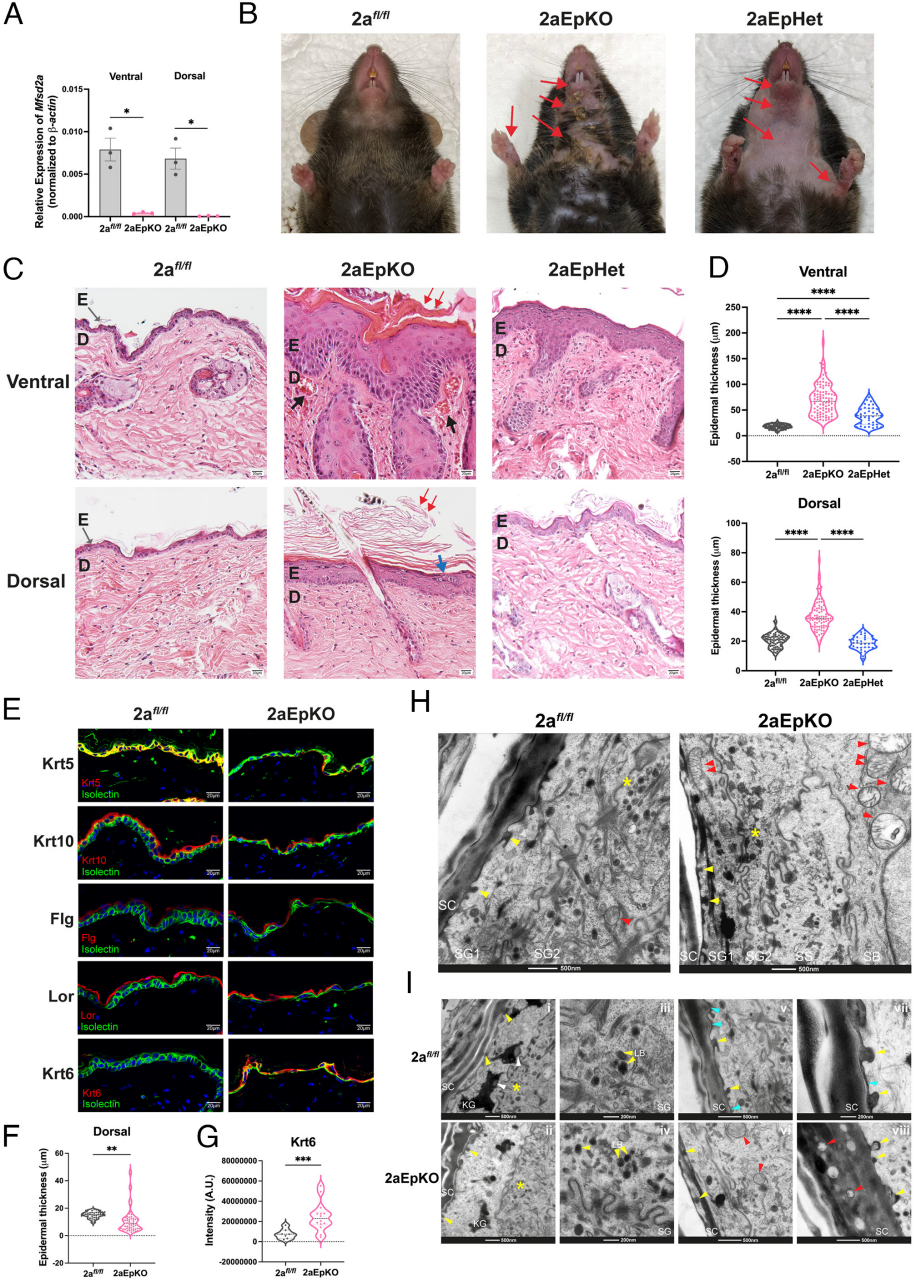
Inducible postnatal Mfsd2a deficiency leads to transient dermatitis with defects in desquamation. (*A*) mRNA expression of *Mfsd2a* in 2aEpKO and 2a*^fl/fl^* control ventral and dorsal epidermis 13 d after tamoxifen treatment. 2a*^fl/fl^*, *n* = 3; 2aEpKO, *n* = 3. (*B*) Photos of mice at day 17 post tamoxifen treatment. 2aEpKO mice developed dermatitis at areas indicated by red arrows, while 2a*^fl/fl^* controls appeared normal. 2aEpHet mice developed alopecia with dermatitis at areas indicated by red arrows. 2a*^fl/fl^*, *n* = 6; 2aEpKO, *n* = 6; 2aEpHet, *n* = 3. (*C*) Representative image of H&E stained skin taken from the chest and back of 2a*^fl/fl^* controls, 2aEpKO, and 2aEpHet mice. 2aEpKO mice developed epidermal hyperplasia and hyperkeratosis (red arrows) with dilated blood vessels (black arrows) in the dermis layer of the chest. Parakeratosis (blue arrow) was observed in 2aEpKO back epidermis. E, epidermis; D, dermis. (Scale bar, 20 µm.) (*D*) Violin plot showing epidermal thickness (µm) measurements in chest and back of 2a*^fl/fl^* controls, 2aEpKO, and 2aEpHet. 2a*^fl/fl^* controls (*n* = 6), 2aEpKO (*n* = 6), and 2aEpHet (*n* = 3). 4 measurements were made per field, 3 to 4 fields were quantified per biological replicate. One-way ANOVA with Dunnett’s T3 multiple comparison test. *P < 0.05; ****P < 0.0001. Dorsal skin harvested from 2a*^fl/fl^* controls and 2aEpKO at day 13 post tamoxifen induction for subsequent panels (*E–I*). (*E*) Immunohistochemical staining of indicated epidermal differentiation markers. Krt5 (basal keratinocytes), Krt10 (suprabasal keratinocytes), Filaggrin (Flg, granular keratinocytes), and Loricrin (Lor, terminally differentiated corneocytes). Krt6 (marker for activated keratinocytes). Isolectin (green) marks basal layer and Hoechst (blue) is a nuclei stain; 2a*^fl/fl^*, *n* = 3; 2aEpKO, *n* = 5. (Scale bar, 20 µm.) (*F*) Violin plot showing epidermal thickness (µm) measurements of dorsal skin of 2a*^fl/fl^* controls (*n* = 5) and 2aEpKO (*n* = 7). 4 measurements were made per field, 3 to 4 fields were quantified per biological replicate. Unpaired *t* test with Welch’s correction, **P < 0.01. (*G*) Violin plot showing quantification of Krt6 expression in dorsal epidermis of 2a*^fl/fl^* controls (*n* = 3) and 2aEpKO (*n* = 4). Unpaired *t* test with Welch’s correction, ***P < 0.001. (*H*) Transmission electron microscopy images of the dorsal epidermis of 2a*^fl/fl^* control (*n* = 17 fields) and 2aEpKO (*n* = 17 fields). Mitochondria (red arrowheads) are normally located in the lower granular keratinocyte layer (“SG2”) of 2a*^fl/fl^*control. Mitochondria are found in the upper granulosum layer (“SG1”) in 2aEpKO. Lamellar bodies (LB) (yellow asterisk) are observed in both 2aEpKO and 2a*^fl/fl^* control epidermis. (Scale bar, 500 nm.) (*I*) Transmission electron microscopy images of 2a*^fl/fl^* control (*n* = 15 fields) and 2aEpKO (*n* = 16 fields). Budding of LB into the stratum corneum (SC) layer are observed in 2aEpKO and 2a*^fl/fl^* control (panels *i*, *ii*, *v*, *vi*, *vii*, and *viii*, yellow arrowheads). However, distinct lipid lamellae (panels *v* and *vii*, blue arrowheads) are observed only in controls but not 2aEpKO. Lipid droplets (LD) are associated with keratohyalin granules (KG) in controls (panel *i*, white arrowheads), KGs are largely devoid of LD in 2aEpKO (panel *ii*). Retainment of LBs (panel *vi*, yellow arrows) and mitochondria (panel *viii*, red arrowheads) were also observed in 2aEpKO SC but not in control SC, consistent with desquamation defects. (Scale bar, 200 nm and 500 nm.)

To study the function of Mfsd2a specifically in the epidermis, we generated an epidermis-specific Mfsd2a deletion model using KRT5-CreER^T2^ (51) (Mfsd2a*^fl/fl^*Krt5-CreER^T2^, hitherto named 2aEpKO). We chose this particular cre driver because it is expressed in the basal cell layer that allows for inducible and irreversible *Mfsd2a* deletion resulting in the continual development of suprabasal keratinocytes that are deficient in Mfsd2a. Tamoxifen treatment of 2aEpKO mice resulted in *Mfsd2a* deficiency in both the ventral and dorsal epidermis (Fig. 2*A*). 17 d post tamoxifen treatment to delete *Mfsd2a*, 2aEpKO mice appeared dermatitic at the mouth, throat, arms and paws relative to Mfsd2a floxed(2a*^fl/fl^*) controls (Fig. 2*B*). Consistent with the appearance of dermatitis, 2aEpKO developed hyperplasia at both the basal and suprabasal layers of the skin with hyperkeratosis, while skin of 2a*^fl/fl^* control mice appeared normal with a thin and compact epidermis (Fig. 2 *C* and *D*). Importantly, mice with inducible deletion of one *Mfsd2a* allele, Mfsd2a*^fl/+^*Krt5-CreER^T2^ (2aEpHet) developed an intermediate phenotype with alopecia at the chest, throat, and paws and mild dermatitis at these areas (Fig. 2*B*). Histological analysis of the skin indicated that 2aEpHet mice developed a milder hyperplasia at the ventral and dorsal region relative to 2aEpKO (Fig. 2 *C* and *D*). Interestingly, hyperplasia and hyperkeratosis at the different skin depots examined from 2aEpKO and 2aEpHet mice occurred with differing severities, with the ventral region being the most affected (Fig. 2 *C* and *D*). A more severe epidermal phenotype on the chest of mice could be a result of increased mechanical stress and expected for a region in constant contact with cage bedding. To gain a more thorough understanding of the temporal development of epidermal changes in 2aEpKO mice, we performed a series of histological analyses at various time points posttamoxifen treatment (*SI Appendix*, Fig. S3 *A* and *B*). The ventral epidermis of 2aEpKO became hyperplasic at 13 d post tamoxifen treatment, while the dorsal epidermis appeared thin with loosely attached cornified sheets at the same time point before becoming hyperkeratotic by day 17 (*SI Appendix*, Fig. S3 *A* and *B*). Importantly, while the dermatitic phenotype appeared to have resolved in 2aEpKO mice by day 28 post tamoxifen treatment, mild hyperplasia, and hyperkeratosis, in addition to parakeratosis was still observed (*SI Appendix*, Fig. S4). Taken together, these data indicate that induced Mfsd2a deficiency results in keratinocyte activation and proliferation with transient dermatitis.

To understand why *Mfsd2a* deficiency led to these phenotypes, we examined 2aEpKO dorsal epidermis at day 13 post tamoxifen treatment prior to development of hyperplasia (Fig. 2 *E* and *F*). Immunohistochemical staining of epidermal differentiation markers was performed to determine if the differentiation state of 2aEpKO epidermis was altered. The markers used were Keratin-5 (Krt5) for basal cell layer, Keratin-10 (Krt10) for suprabasal keratinocytes, Filaggrin (Flg) for the granular layer and Loricrin (Lor) for cells comprising the corneum layer, and Krt6 as a marker for activated keratinocytes found in epidermal pathologies such epidermal barrier dysfunctional states (52, 53). Although this marker analysis indicated the presence of all the cell layers of the epidermis in 2aEpKO skin (Fig. 2*E*), Keratin-6 (Krt6) expression was observed in 2aEpKO but not 2a*^fl/fl^* controls (Fig. 2 *E* and *G*), indicative of keratinocytes being in an activated and proliferative state. Consistent with this conclusion, increased Krt6 expression and Ki67 positive keratinocytes was observed in 2aEpKO but not 2a*^fl/fl^* controls across all the timepoints posttamoxifen treatment that were examined (*SI Appendix*, Fig. S3 *C*–*E*).

Transmission electron microscopy images of 2a*^fl/fl^* and 2aEpKO epidermis showed retainment of mitochondria and LB in the upper granulosum layer and stratum corneum (SC), respectively, only in 2aEpKO epidermis (Fig. 2 *H* and *I*), in agreement with defects in desquamation. Although LB in the SC layer were observed in both 2aEpKO and 2a*^fl/fl^* control epidermis, distinct lipid lamellae at the SC were observed only in controls but not 2aEpKO (Fig. 2*I*). Moreover, while LD were found to be associated with keratohyalin granules (KG) in controls, KGs were largely devoid of LD in 2aEpKO (Fig. 2*I*). Taken together, these findings indicate that inducible Mfsd2a deficiency in epidermal keratinocytes leads to defects in keratinocyte desquamation.

**Fig. 3.**
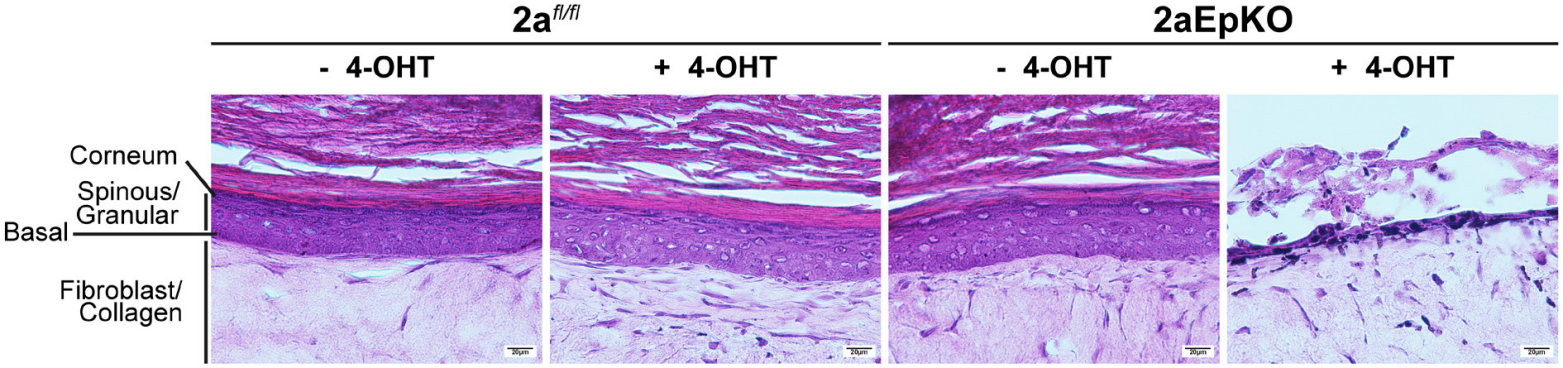
Mfsd2a is essential for epidermal stratification in vitro. Primary keratinocytes isolated from the same mouse (2a*^fl/fl^* and 2aEpKO) was split into two groups, treated and untreated with 4-hydroxytamoxifen (4-OHT). Representative images of H&E stained stratified epidermal cultures derived from 2a*^fl/fl^* and 2aEpKO keratinocytes after 14 d of air exposure. 2a*^fl/fl^*, *n* = 4 mice; 2aEpKO, *n* = 5 mice. (Scale bar, 20 µm.)

The observation that inducible deletion of *Mfsd2a* in 2aEpKO mice resulted in transient dorsal epidermis thinning indicative of reduced keratinocyte proliferation and differentiation that ultimately resolves to become hyperproliferative (*SI Appendix*, Fig. S3 *A* and *B*) suggests that *Mfsd2a* has a cell autonomous role in keratinocyte proliferation and differentiation. To test this idea, we made use of an in vitro organotypic epidermis culture system (54). Briefly, this system involves a collagen bed containing primary mouse dermal fibroblasts as mimetic of the skin dermis. Primary mouse keratinocytes are overlaid on the collagen/fibroblast bed and cultured in media for 4 d, after which epidermal stratification was induced by exposing the keratinocyte layer to the air–liquid interface with serum containing media that endogenously has LPCs (*SI Appendix*, Fig. S5*A*). Both 2a*^fl/fl^* and 2aEpKO keratinocytes not treated with 4-hydroxytamoxifen were able to stratify into an epidermis (Fig. 3, *SI Appendix*, Fig. S5*B*). In contrast, 4-hydroxytamoxifen treated 2aEpKO keratinocytes, but not 2a*^fl/fl^* keratinocytes, failed to stratify into an epidermis (Fig. 3, *SI Appendix*, Fig. S5*B*). Epidermal differentiation markers were expressed in the expected layers in 2a*^fl/fl^* and untreated 2aEpKO indicative of proper epidermal stratification (*SI Appendix*, Fig. S5*C*). These results indicate that Mfsd2a plays an essential cell autonomous role in keratinocyte proliferation and differentiation in this in vitro experimental setup.

### Mice With Conventional Deficiency of Mfsd2a Have a Defect in Epidermal Desquamation.

Whole body conventional deletion of Mfsd2a (2aKO) does not develop obvious epidermal defects as we found for inducible adult deletion of Mfsd2a (2aEpKO) (Fig. 2). However, closer examination of the skin by histological analysis revealed that 2aKO exhibited hyperkeratosis with appearance of thickened sheets of dead corneocytes relative to the compact SC observed in wildtype (WT) controls in addition to the retention of visible nuclei (parakeratosis) in the SC (*SI Appendix*, Fig. S6 *A* and *B*). Taken together, these phenotypes are consistent with defects in desquamation in 2aKO mice. Immunohistochemical staining of epidermal differentiation markers indicated Krt5 expression is retained in suprabasal keratinocytes of 2aKO (*SI Appendix*, Fig. S6 *C* and *D*), which suggests a defect in the commitment of basal cell differentiation into spinous cells. Moreover, Krt6 expression was detected in 2aKO but not WT epidermis (*SI Appendix*, Fig. S6 *C* and *E*), indicative of 2aKO keratinocytes being in an activated and proliferative state. Together, these data are supportive that 2aKO epidermis exhibit hyperkeratosis and desquamation defects similar to 2aEpKO mice.

### Mfsd2a Deficiency Results in Dysregulation of Cornified Envelope Development.

**Fig. 4.**
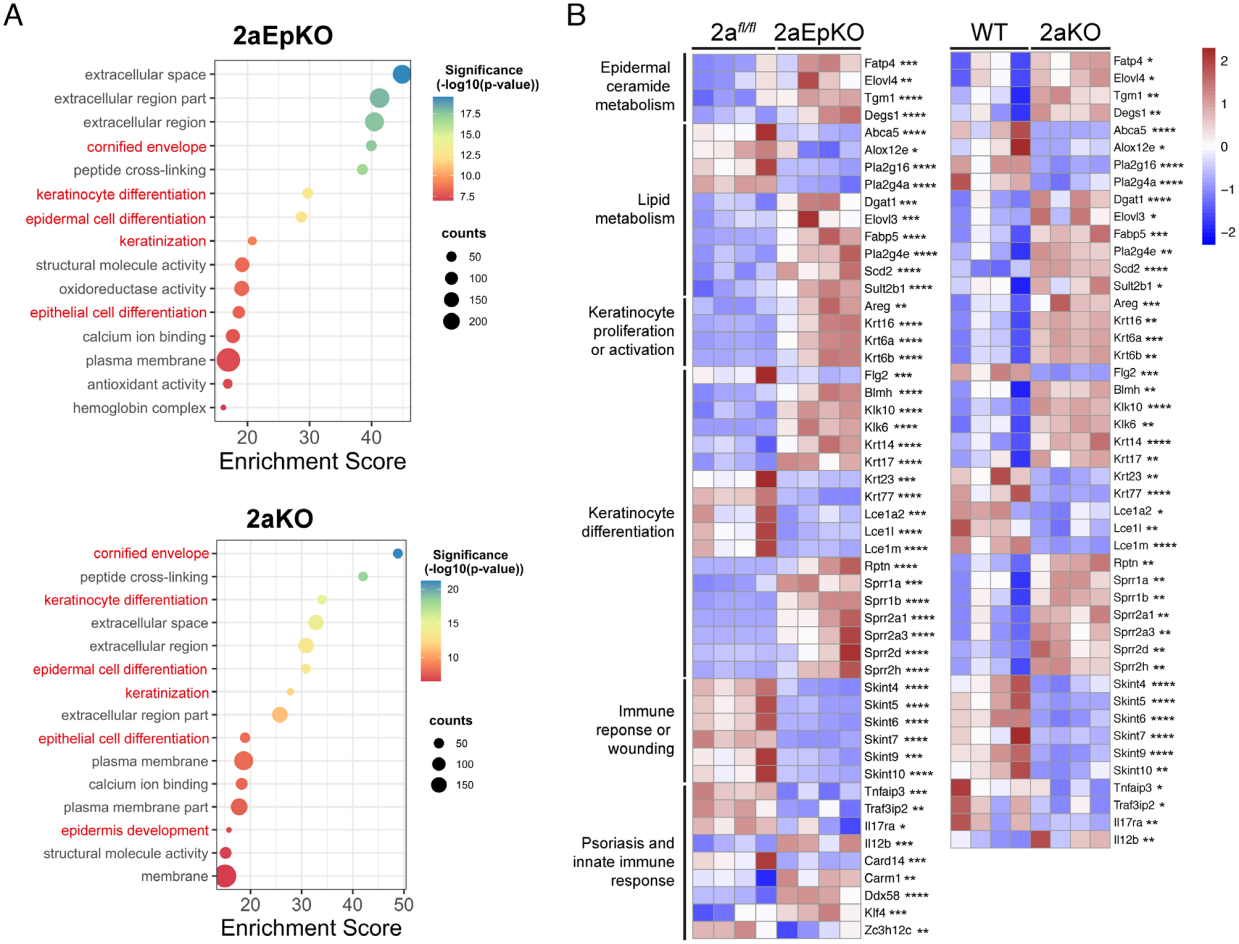
Mfsd2a deficiency results in changes in gene expression indicative of defective keratinocyte differentiation. RNA-seq analysis of 2aEpKO and 2aKO epidermis versus their respective controls. Dorsal epidermis of 2aEpKO mice harvested at day 13 post tamoxifen induction. (*A*) Bubbleplots representing the top 15 significantly up- or down-regulated Gene Ontology Terms in 2aEpKO or 2aKO relative to their respective controls. Genes with a fold change of ≥2 or ≤−2 and a *P* < 0.05 were used for pathway analysis, and pathways of interest are labeled in red text. (*B*) Heatmaps represent changes between 2aEpKO or 2aKO versus their respective controls for genes constituting pathways of high importance in epidermal biology. The color bar indicates z-score transformation on median ratio normalized counts. Wald test, *P < 0.05; **P < 0.01; ***P < 0.001; ****P < 0.0001. For all panels, *n* = 4 per genotype; biological replicates.

To provide a higher-resolution analysis of epidermal differentiation, transcriptomic analysis by RNA-sequencing was carried out on isolated epidermis from 2aEpKO at day 13 post tamoxifen treatment, 2aKO and their respective controls. We found that 2aEpKO and 2aKO had 3,216 and 1,396 significantly upregulated differentially expressed genes (DEGs), respectively, and 2,855 and 1,331 significantly downregulated DEGs, respectively. Pathway analysis of DEGs between 2aEpKO versus 2a*^fl/fl^* and 2aKO versus WT identified alterations in cornified envelope development and keratinocyte differentiation as part of the top 15 significant Gene Ontology terms (Fig. 4*A*).

Genes associated with keratinocyte differentiation and proliferation were found to be increased in 2aEpKO and 2aKO relative to their respective controls (Fig. 4*B*). In particular, the upregulation of Krt6a was observed in both Mfsd2a deficiency models, consistent with increased Krt6 protein expression in 2aEpKO (Fig. 2 *E* and *G*) and 2aKO (*SI Appendix*, Fig. S6 *C* and *E*). Lce1s and Sprr2a1, which are involved in cornified envelope development, were significantly reduced in 2aEpKO and 2aKO relative to their respective controls (Fig. 4*B*). Both Fatp4 and Tgm1, proteins essential for acylceramide synthesis and crosslinking of acylceramide to the cornified layer, where mutations in these genes lead to ichthyosis (55, 56), were upregulated in 2aEpKO and 2aKO relative to their respective controls (Fig. 4*B*), possibly representing an adaptive mechanism to maintain acylceramide synthesis in the absence of Mfsd2a. Moreover, genes associated with fatty acid metabolism were increased in 2aEpKO and 2aKO relative to their respective controls (Fig. 4*B*). Other major pathway changes common to 2aEpKO and 2aKO epidermis were genes associated with immune responses, psoriasis, and wound healing. In sum, these data indicate that changes in the epidermal transcriptome of 2aEpKO mirror changes observed in 2aKO (*SI Appendix*, Fig. S7) and that Mfsd2a deficiency results in transcriptional responses consistent with major changes in cornified envelope development and desquamation.

### LPC Uptake Into the Epidermis is Dependent On Mfsd2a Expression.

**Fig. 5.**
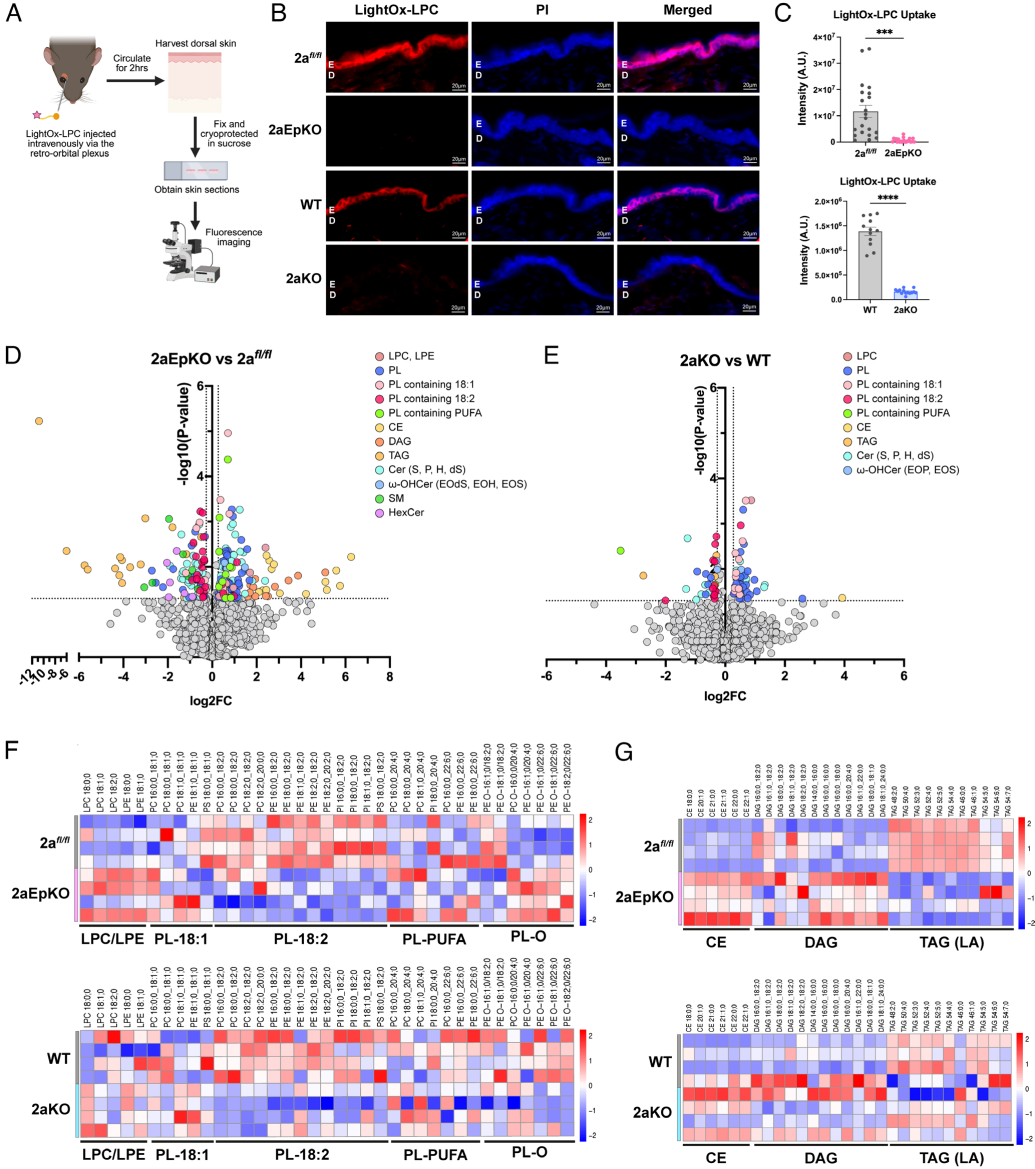
Mfsd2a is required for LPC uptake into epidermis and for normal maintenance of the epidermal phospholipidome. (*A*) Schematic of LightOx-LPC administration. LightOx-LPC was injected intravenously in 2aEpKO, 2aKO, and their respective controls and allowed to circulate for 2 h. Dorsal skin was fixed and sectioned for fluorescence imaging. (*B*) Representative image of LightOx-LPC (red) accumulation in the epidermis of 2aEpKO, 2aKO, and their respective controls. Propidium Iodine (PI, blue) is a nuclei stain. *n* = 3 per genotype. E, epidermis; D, dermis. (Scale bar, 20 µm.) (*C*) Scatterplot with bars showing quantification of LightOx-LPC fluorescence in (*B*). Individual points indicate each field quantified and bar charts for mean ± SE. Unpaired *t* test with Welch’s correction, ***P < 0.001; ****P < 0.0001. Volcano plots of lipidomic analysis showing significant lipid changes in 2aEpKO (*D*) and 2aKO (*E*) epidermis versus their respective controls. Epidermal lipid species represented as mol% of phospholipids (PL), sphingolipids (SL), or neutral lipids (NL). Colored dots indicate lipid species that are significantly different in 2aEpKO or 2aKO epidermis relative to their respective controls with a fold change of >1.2 or <−1.2. (*F*) Heatmap representation of log_2_-transformed fold change in phospholipids of 2aEpKO and 2aKO relative to their respective controls. The color bar indicates z-score transformation on mol% PL. (*G*) Heatmap representation of log_2_-transformed fold change in NL of 2aEpKO and 2aKO relative to their respective controls. The color bar indicates z-score transformation on mol% NL. For panels (*D*–*G*) in this figure, *n* = 4 per genotype.

Since the epidermis is avascular, the expression of Mfsd2a in differentiated keratinocytes of the epidermis raises the important question about the origin of the LPCs that are transported by Mfsd2a. To begin to address this question we tested whether plasma-derived LPCs can reach the suprabasal cells of the epidermis that express Mfsd2a. We hypothesized that plasma-derived LPCs would diffuse from the dermis vasculature to the epidermis and be taken up in an Mfsd2a-dependent fashion. To test this idea, we utilized a fluorescently labeled LPC named LightOx-LPC that we previously demonstrated to be specifically transported by Mfsd2a (42). LightOx-LPC was delivered intravenously into 2aEpKO, 2aKO, and their respective controls and allowed to circulate for 2 h before harvesting dorsal skin for examination by fluorescent microscopy (Fig. 5*A*). Epidermis of 2a*^fl/fl^* and WT mice, but not 2aEpKO and 2aKO, accumulated LightOx-LPC (Fig. 5 *B* and *C*), indicating that plasma-derived LPC uptake by keratinocytes in vivo is Mfsd2a dependent.

### Mfsd2a Deficiency Alters the Epidermal Phospholipidome.

Given that Mfsd2a is functionally mediating LPC transport into the epidermis (Fig. 5 *B* and *C*), we next sought to determine the consequences of Mfsd2a deficiency on the lipid composition of the epidermis. To carry this out, we quantified changes in the epidermal lipidome in response to Mfsd2a deficiency with a focus on noncovalent lipids that constitute the lipids produced by keratinocytes but are not cross-linked to the cornified layer. We utilized epidermal sheets from 2aEpKO at day 13 post tamoxifen treatment, 2aKO and their respective controls that were enzymatically dissociated from their underlying dermis and sebaceous glands. Lipidomic analysis was carried out using a shotgun approach, which allowed for wide coverage of phospholipids and skin ceramides with absolute quantification.

Notably, phospholipid species containing-linoleic acid (PL-18:2), one of the most abundant fatty acid species found in epidermal phospholipids, was decreased by 15% in 2aEpKO (Fig. 5 *D* and *F* and *SI Appendix*, Fig. S8*A*) and 13% in 2aKO (Fig. 5 *E* and *F*, *SI Appendix*, Fig. S8*B*) relative to their respective controls. Additionally, LPC and lysophosphatidylethanolamine (LPE) species, which are products of phospholipase activity, were increased by 60 and 28% respectively in 2aEpKO relative to 2a*^fl/fl^* controls (Fig. 5 *D* and *F* and *SI Appendix*, Fig. S8*A*) but were mostly found to be unchanged in 2aKO (Fig. 5 *E* and *F* and *SI Appendix*, Fig. S8*B*). Plasma levels of LPC and LPE species in 2aEpKO were similar relative to 2a*^fl/fl^* controls, with the exception of a 21% increase in LPE-18:0 in 2aEpKO (*SI Appendix*, Fig. S8 *C* and *D*), indicating that epidermal Mfsd2a does not influence plasma LPC/LPE concentrations. It is known that epidermal triglyceride (TAG) containing linoleic acid is utilized by Pnpla1 to generate acylceramides (13, 14). Remarkably, the major TAG species containing linoleic acid were decreased by 79% in 2aEpKO relative to 2a*^fl/fl^* controls with a reciprocal 1.65-fold increase in DAG species devoid of linoleic (Fig. 5 *D* and *G* and *SI Appendix*, Fig. S8*E*), a potential compensatory response to Mfsd2a deficiency that is expected to be important for maintaining epidermal barrier function. Indeed, a few minor changes in ceramide containing lipids, such as increased acylceramides EOS and EOH by 26% and 46% respectively in 2aEpKO (Fig. 5*D* and *SI Appendix*, Fig. S8*F*), were observed with Mfsd2a deficiency. Although neutral lipids (NL) were mostly unchanged in keratinocytes of 2aKO relative to WT mice (Fig. 5 *E* and *G* and *SI Appendix*, Fig. S8*G*), acylceramide EOS was decreased by 18% in 2aKO, while EOP was increased by 41% in keratinocytes of 2aKO mice (Fig. 5*E* and *SI Appendix*, Fig. S8*H*). In total, this analysis indicates that while induced deletion of Mfsd2a in 2aEpKO resulted in changes in NL and acylceramides that were not observed in 2aKO, both 2aEpKO and 2aKO exhibited a deficiency in phospholipid pools containing linoleic acid (18:2). This finding is significant because LPC-18:2 is the major polyunsaturated LPC found in mouse and human plasma (34, 35, 57, 58) and points to LPC-18:2 as important for maintaining epidermal phospholipid composition and differentiation via uptake by Mfsd2a.

### LPC18:1 and LPC18:2 Uptake by MFSD2A Promotes Human Primary Keratinocyte Differentiation.

**Fig. 6.**
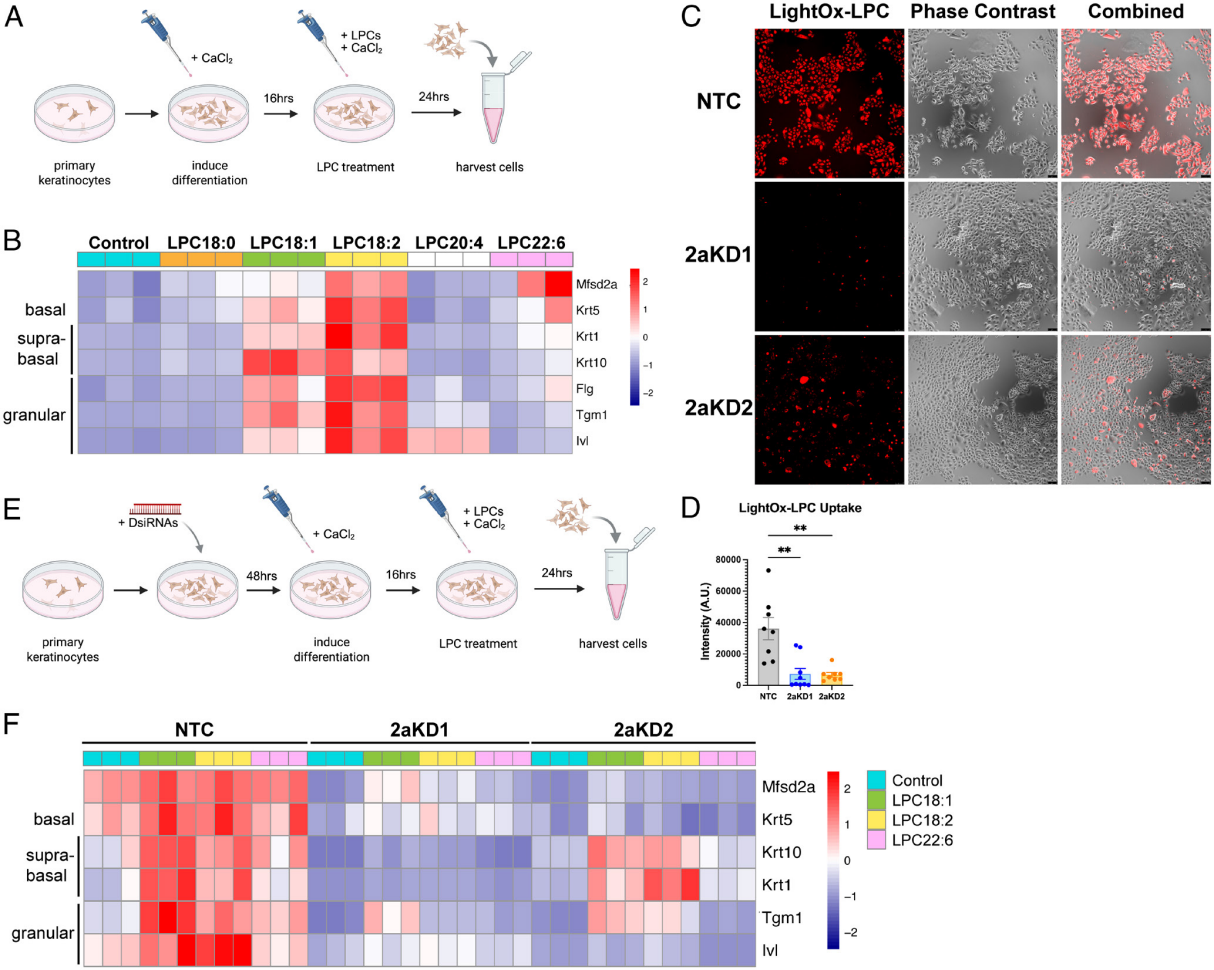
LPC18:1 and LPC18:2 enhance human primary keratinocyte differentiation in a MFSD2A-dependent manner. (*A*) Schematic diagram of experimental setup for primary keratinocyte differentiation with the addition of LPCs. (*B*) Heatmap representation of fold changes in mRNA of skin differentiation markers in LPC treated versus control keratinocytes. (*C*) A functional test of MFSD2A knockdown using LightOx-LPC to measure MFSD2A transport activity in nontargeting DsiRNA (NTC) or in cells treated with two different *MFSD2A* DsiRNA (2aKD1 and 2aKD2). Representative image of LightOx-LPC (red) fluorescence and phase-contrast images in human primary keratinocytes transfected cells with DsiRNA. (*D*) Scatterplot with bars showing quantification of uptake of LightOx-LPC fluorescence in (*C*); One-way ANOVA with Dunnett’s T3 multiple comparison test. **P < 0.01. (*E*) Schematic diagram outlining differentiation of *MFSD2A*-knockdown primary keratinocytes with the addition of LPCs. (*F*) Heatmap representation of fold changes of skin differentiation markers in different treatment group versus NTC keratinocytes. The color bar indicates z-score transformation of fold change values in panels (*B* and *F*). *n* = 3 per treatment, technical replicates.

To determine if LPC-18:2 uptake by MFSD2A is important for human keratinocyte differentiation, human primary keratinocytes were used for functional studies. Terminal differentiation of human keratinocytes in vitro can be achieved by treating keratinocytes with a high concentration of calcium for 10 to 14 d (59, 60). Given that calcium is a potent inducer of keratinocyte differentiation, these cells were treated with LPCs following a short pretreatment with calcium to induce their differentiation (Fig. 6*A*). A comparison of LPCs of different chain lengths and unsaturation states (*SI Appendix*, Fig. S9*A*) revealed that in addition to LPC-18:2, LPC-18:1 was also able to induce keratinocyte differentiation, as seen by increased expression of suprabasal (Krt1, Krt10) and granular differentiation markers (Flg, Tgm1, and Ivl) (Fig. 6*B* and *SI Appendix*, Fig. S9*B*) relative to nontreated control. In contrast to LPC-18:1, unesterified fatty acid 18:1 was not able to induce keratinocyte differentiation (*SI Appendix*, Fig. S9*C*). Knockdown of *MFSD2A* in primary human keratinocytes was induced using the duplex RNA knockdown strategy using 2 different DsiRNAs. Utilizing the MFSD2A activity probe LightOx-LPC in cells treated with two different DsiRNAs designated 2aKD1 and 2aKD2 showed reduced uptake of LightOx-LPC relative to nontargeting control (NTC) (Fig. 6 *C* and *D*). Knockdown of *MFSD2A* mRNA was confirmed by qRT-PCR (*SI Appendix*, Fig. S9*D*). Following Mfsd2a knockdown, keratinocytes were induced to differentiate with calcium, then cotreated with calcium and LPCs (Fig. 6*E*). Knockdown of *MFSD2A* in keratinocytes reduced their ability to differentiate in response to LPCs relative to NTC (Fig. 6*F* and *SI Appendix*, Fig. S9*D*), which indicates that LPC uptake by keratinocytes in vitro is Mfsd2a dependent and can enhance keratinocyte differentiation.

## Discussion

The epidermis is a complex multilayered organ that undergoes regulated proliferation and differentiation of precursor basal cells into specialized suprabasal keratinocytes that synthesize and secrete LB containing phospholipids that are essential for barrier function. It is known that the epidermis is completely reliant on dietary linoleic acid for acylceramide synthesis, but whether the epidermis is also reliant on plasma-derived phospholipid has not been explored. One class of plasma phospholipid, LPC is a prime candidate for serving such a function in skin since it is transported into cells by Mfsd2a (27, 28, 31, 34–39, 41, 42, 58, 61, 62). Here, we demonstrated that Mfsd2a, a sodium-dependent LPC transporter, is expressed in both suprabasal and differentiated keratinocytes where it mediates plasma-derived LPC uptake and is critical for maintaining phospholipid-containing linoleic acid pools in the epidermis and for normal desquamation.

Similar to dietary linoleic acid deficiency in rodents (63, 64), inducible deletion of Mfsd2a in the adult epidermis of mice (2aEpKO) led to the development of dermatitis. Our lipidomic analysis of isolated epidermis revealed that 2aEpKO mice resulted in significant reductions in phospholipids and triglycerides containing linoleic acid (PL-18:2, TAG-18:2) compared to 2a*^fl/fl^* controls. It is notable that these reductions in linoleic acid occurred in conditions in which mice were fed diets with sufficient linoleic acid, indicating that Mfsd2a mediated LPC uptake into the epidermis is uniquely important for maintaining the epidermal lipidome. The moderate decrease in PL-18:2 in the epidermis of 2aEpKO that we report here could be an underestimation because total epidermis and not the stratum granulosum was analyzed. The decrease in TAG-18:2 pools with a reciprocal increase in DAG pools depleted in linoleic acid in epidermis of 2aEpKO is suggestive of increased Pnpla1 activity, the rate-limiting acyltransferase that utilizes TAG-18:2 to synthesize acylceramides (13, 14). This particular depletion in TAG-18:2 in 2aEpKO that was not observed in 2aKO mice is suggestive of an adaptive response to the acute inducible Mfsd2a deficiency that at later timepoints post-Mfsd2a deletion leads to a severe but transient dermatitis. Nonetheless, both 2aEpKO and 2aKO both have a deficiency in phospholipids containing 18:2, defects in epidermal desquamation and highly similar transcriptional profiles in their keratinocytes. It is also notable that inducible haploinsufficiency in 2aEpHET mice resulted in the development of an intermediate epidermal phenotype, supporting the conclusion that Mfsd2a is rate-limiting for epidermal LPC uptake and in maintaining normal epidermal structure and health. No overt skin phenotypes have been reported for patients with inactivating variants in MFSD2A. However, based on our findings that 2aKO mice did not display gross anatomical changes in skin like the dermatitic phenotype observed in inducible deficiency of *Mfsd2a* (2aEpKO) (Fig. 2 and *SI Appendix*, Fig. S6), skin biopsy analysis would likely be required in MFSD2A patients to identify potential epidermal defects such as the defects in desquamation seen in 2aKO mice (*SI Appendix*, Fig. S6).

A common theme among tissues where Mfsd2a is expressed is that they have a high demand for phospholipid and/or essential fatty acids (e.g. DHA, linoleate) such as brain, eye, lung, liver, and epidermal keratinocytes. Switzer and Eder originally speculated that LPCs in plasma likely serve as phospholipid precursors for renewal of membrane phospholipids (65). This prophetic idea is supported by the observation that one of the most abundant LPCs found in human and rodent plasma is LPC-18:2 (34, 35, 57, 66), raising the possibility that plasma LPC-18:2 is a quantitatively important source of phospholipid for the epidermis. The epidermis/skin is the largest organ in the body and expresses a high level of *MFSD2A* (Fig. 1*A*). A study quantifying the fatty acid composition of phospholipids in human epidermal layers found that phospholipid composition shifts from a 16:0 and 18:1 majority in the basal and spinous layers to an increase in 18:2 in the granulosum layer (67) where LBs are synthesized and a layer in which Mfsd2a is expressed the greatest among suprabasal keratinocytes. These findings align with our analysis of Mfsd2a function in the epidermis.

Emerging evidence indicates that dynamic changes in the lipidome of human keratinocytes are associated with differentiation state, with phosphatidylcholine species among some of the top lipid associations that include PC containing 18:2 (68). Our findings that LPC-18:2 enhanced calcium-induced differentiation of human keratinocytes raises the possibility that LPC-18:2 uptake by MFSD2A modulates the activity of the calcium-release calcium activated channel 1, Orai1, previously shown to have a role in calcium active keratinocyte differentiation (69). Given that the epidermis undergoes continual cell proliferation, differentiation, and lipid secretion of phospholipid-rich LBs highlights that the epidermis has a high demand for phospholipids, the uptake of phospholipid (as LPC) might be coordinated with proper epidermal differentiation. Our findings fit reasonably well with a model in which plasma LPCs serve a purpose as a precursor for PC biosynthesis containing PUFAs through the action of epidermal LPCAT activity to support synthesis of phospholipid-rich LBs and possibly as a source for linoleic acid stored in TAG for acylceramide biosynthesis. Interestingly, skin diseases such as Harlequin Ichthyosis and Chanarin-Dorfman syndrome (ABHD5/CGI58 deficiency) are associated with absent or reduced LBs, abnormal LB contents, or incomplete LB secretion, that ultimately leads to defects in lipid lamellar matrix composition and diminished barrier function (70–72). Given our findings that LPCs are an important source for phospholipid and linoleic acid in keratinocytes though transport via Mfsd2a, it will be important to consider whether LPCs can be used as a therapeutic agent to treat skin diseases such as atopic dermatitis and psoriasis.

## Materials and Methods

### Mouse Models.

Experimental protocols involving mice were approved by SingHealth Institutional Animal Care and Use Committee under reference number 2023/SHS/1816. Adult mice were anesthetized with a combination of ketamine (20 mg/kg body weight) and xylazine (2 mg/kg body weight) for all experiments. Mfsd2a lineage tracing mouse line (designated as Mfsd2a^ERT2cre^) was generated by genetically knocking in tamoxifen inducible cre (ERT2cre) into exon 1 of the Mfsd2a gene. The Mfsd2a^ERT2cre^ line was then crossed to a cre recombinase inducible TdTomato reporter line [B6;129S6-Gt(ROSA)26Sor*^tm9(CAG-tdTomato)Hze^*/J, The Jackson Laboratory] (73). Mfsd2a whole-body KO (2aKO) mice were generated as described previously (39). Mfsd2a floxed mice (2a*^fl/fl^*) were generated as described previously (28). Tamoxifen-inducible epidermis-specific knockout mice (2aEpKO) were generated by crossing 2a*^fl/fl^* with Krt5-cre/ERT2 driver [B6N.129S6(Cg)-Krt5*^tm1.1(cre/ERT2)Blh^*/J, The Jackson Laboratory] (51). All mice were housed in colony cages on a 12 h light/12 h dark cycle with controlled humidity and temperature at 23 °C. 2aKO and wild-type controls were fed ad libitum with a high energy diet 5LJ5 (PicoLab) with a total of 11% fat, while all other animals were fed ad libitum on a normal chow diet (Global 18% Protein Rodent Diet from Harlan, Envigo) and have free access to water. Pups were weaned at 3 wk of age. Both male and female mice were used in all experiments. Tamoxifen preparation and induction protocol as described in *SI Appendix*, *Supplementary Methods*.

### Histological Studies.

To prepare skin sections, skin was isolated from the chest (ventral) and back (dorsal) of deeply anesthetized mice and fixed in 4% paraformaldehyde (PFA) in PBS at 4 °C overnight. Skin sections (5 and 12 µm) from paraffin blocks and frozen blocks respectively were prepared for downstream H&E staining, immunohistochemical, or immunofluorescence analysis. Epidermal stratified cultures were carried out as described by Ikuta et al. (54) with modifications. Epidermal sheets and collagen beds were fixed in 4% PFA in PBS at 4 °C overnight and processed as before. The following antibodies were used: RFP (1:100), cytokeratin 5 (1:200), cytokeratin 10 (1:200), Filaggrin (1:200), Loricrin (1:100), cytokeratin 6A (1:100), Ki-67 (1:100), Pdgfra (1:100), and Isolectin GS-IB4 (1:100). Images were obtained using the BX53 Light Microscope (Olympus) or LSM710 Confocal Microscope (Carl Zeiss) where indicated. Additional details described in *SI Appendix*, *Supplementary Methods*.

### Isolation of Epidermal Sheets for Lipidomics Analysis and RNA-sequencing.

Dorsal skin explants were harvested from day 13 posttamoxifen induced 2a*^fl/fl^* or 2aEpKO mice or 2 mo old WT or 2aKO mice as described by Poumay et al. with modifications (74). After scraping off the underlying subcutaneous fat, skin explants were cut into thin 5 mm strips and allowed to float epidermis side up on 10U/ml Dispase II (Gibco) in calcium- and magnesium-free HBSS at 37 °C for 1 to 1.5 h. Epidermis was carefully separated from the dermis using fine-tipped forceps, rinsed briefly in cold calcium- and magnesium-free DPBS and weighed, before carrying out a short spin to remove DPBS. Additional details described in *SI Appendix*, *Supplementary Methods*.

### Lipidomic Analysis.

Mass spectrometry-based lipid analysis was performed by Lipotype GmbH (Dresden, Germany). Epidermal sheets were homogenized in phosphate buffered saline to a final concentration of 5 mg/ml and 150 µl was used for extraction using a two-step chloroform/methanol procedure (75). After extraction, the organic phase was transferred to an infusion plate and dried in a speed vacuum concentrator. Samples were analyzed by direct infusion on a QExactive mass spectrometer (ThermoScientific) equipped with a TriVersa NanoMate ion source (Advion Biosciences). Data were analyzed with Lipotype’s in-house developed lipid identification software based on LipidXplorer (76, 77). Data postprocessing and normalization were performed using Lipotype’s in-house developed data management system. Only lipid identifications with a signal-to-noise ratio >5, and a signal intensity 5-fold higher than in corresponding blank samples were considered for further data analysis. Lipid species were normalized to mol% of their respective classes (sphingolipids, phospholipids, or NL) and used for downstream analysis. Additional details described in *SI Appendix*, *Supplementary Methods*. Data available in Dataset S1.

### RNA-Sequencing.

Library preparation and RNA-seq performed by NovogeneAIT (Singapore). 1 µg RNA per sample was used for library preparation using NEBNext® Ultra TM RNA Library Prep Kit for Illumina® (NEB, USA) according to the manufacturer’s instructions, sequenced on the Illumina HiSeq2000 platform and analysis was performed using Partek Flow (version 9). Additional details described in *SI Appendix*, *Supplementary Methods*. DESeq2 normalized counts available in Dataset S1.

### Human Primary Keratinocyte Culture.

The use of deidentified human keratinocyte cell line was approved by National University of Singapore Institutional Review Board under reference number: NUS-IRB-2023-576. Human primary keratinocytes were cultured according to Rheinwald and Green’s method (78). Briefly, keratinocytes were cultured on a feeder layer of lethally irradiated (60 Gy) 3T3-J2 fibroblasts (gift from late Howard Green’s laboratory) in complete FAD medium (79). Upon reaching confluency, the feeder layer was first removed using 0.2 mM EDTA, before the keratinocytes were trypsinized with 0.05% trypsin-EDTA for 5 to 10 min 37 °C. Keratinocytes were expanded once in serum free media (KGM, Lonza) with insulin and keratinocyte supplements on noncoated tissue culture flasks before seeding for experiments. Keratinocytes were treated with 1.2 mM calcium chloride CaCl_2_ for 16 h to induce keratinocyte differentiation prior to LPC treatment. LPCs containing fatty acid of various chain lengths or unsaturation states (5 µM LPC18:0, 25 µM LPC18:1, 25 µM LPC18:2, 25 µM LPC20:4, or 5 µM LPC22:6) or unesterified fatty acid (25 µM FFA18:1) was carried for 24 h in the presence of CaCl_2_ before RNA was harvested using the RNeasy kit (Qiagen) according to the manufacturer’s recommended protocol. To knockdown MFSD2A in keratinocytes, we transfected cells with two different *MFSD2A* DsiRNA (10 nM each of hs.Ri.MFSD2A.13.1 or hs.Ri.MFSD2A.13.2, IDT). In addition, keratinocytes were transfected with 10 nM negative control DsiRNA (NTC) that is nontargeting in humans. After 48 h, keratinocytes were treated with LPCs (25 µM LPC18:1, 25 µM LPC18:2, and 5 µM LPC22:6) and RNA harvested as before. For all experiments, cDNA was synthesized from 1 μg of total RNA using iScript Reverse Transcriptase Supermix (Bio-Rad) according to the manufacturer’s recommended protocol. Quantitative RT-PCR (qRT-PCR) was performed with the SensiFAST SYBR Hi-ROX Kit (Bioline) using the QuantStudio6Pro (Applied Biosystems). Primer information provided in *SI Appendix*, Table S1. Data were normalized to housekeeping gene *RPL13A*. For in vitro transport studies, keratinocytes were treated with 10 µM LightOx-LPC in 12% fatty-acid free BSA for 30mins in 37 °C, 5% CO_2_ incubator. LightOx fluorescence was excited at the UV wavelength (405 nm). Images were obtained using the DMi8 fluorescence microscope (Leica).

### Statistical Analysis.

Statistical analysis was calculated using either unpaired *t* test with Welch’s correction, one-way ANOVA with the Kruskal–Wallis multiple comparison test or Dunnett’s multiple comparison test, or two-way ANOVA with Tukey’s multiple comparison test where stated in the figure legends. All graphs and statistical tests were carried out on GraphPad PRISM and R 3.4.0. A *P* < 0.05 was considered to be significant.

## Supplementary Material

Appendix 01 (PDF)

Dataset S01 (XLSX)

## Data Availability

All study data are included in the article and/or supporting information.
