# Supplementary material for: Mfsd2a is important for maintaining epidermal homeostasis
Source: Proc Natl Acad Sci U S A. 2026 Feb 19;123(8):e2531159123. doi: 10.1073/pnas.2531159123 (PMC12933103; doi:10.1073/pnas.2531159123)
Supplement: Supplementary file 1 — Appendix 01 (PDF) [file pnas.2531159123.sapp.pdf]

PNAS

Supplementary Information for:

Mfsd2a is important for maintaining epidermal homeostasis

Bernice H. Wong[a], Kunal Mishra[a,b], Cheen Fei Chin[a], Dwight L.A. Galam[a], Bryan C. Tan[a], Mei Ding[c,d], Federico Torta[a,c,d], Jacques Behmoaras[a,b], Alvin W.C. Chua[e,f] and David L. Silver[a1]

[a]Signature Research Program in Cardiovascular and Metabolic Disorders, Duke-NUS Medical School, Singapore
[b]Centre for Computational Biology, Duke-NUS Medical School, Singapore
[c]Singapore Lipidomics Incubator (SLING), Life Sciences Institute
[d]Precision Medicine Translational Research Programme and Department of Biochemistry, YLL School of Medicine, National University of Singapore, Singapore
[e]Department of Plastic Reconstructive & Aesthetic Surgery, Singapore General Hospital, 20 College Road, Singapore 169856, Singapore
[f]Musculoskeletal Sciences Academic Clinical Programme, Duke–NUS Medical School, Singapore

This PDF file includes:

Supplementary Methods
Supplementary Tables S1 to S2
Figures S1 to S9
SI References

Other supplementary materials for this manuscript include the following:

Dataset S1

Supplementary Methods

Reagents

Lipids used in this study were LPC18:0 (855775, Avanti Research); LPC18:1 (845875, Avanti Research); LPC18:2 (prepared as described in (1)); LPC20:4 (prepared as described in (1)); and LPC22:6 (prepared as described in (1)). Fluorescently labelled LPC (LightOx-LPC) was a kind gift from Travecta Therapeutics. Antibodies used in this study were: RFP (600-401-379, Rockland); cytokeratin 5 [EP1601Y] (ab193895, Abcam); cytokeratin 10 [EP1607IHCY] (ab194231, Abcam); Filaggrin (905804, BioLegend); Loricrin (905104, Biolegend); cytokeratin 6A (905701, Biolegend); Ki-67 (ab15580, Abcam), Pdgfra (3174S, Cell Signalling) and Isolectin GS-IB4 (I21411, ThermoFisher Scientific). For Immunotistochemical staining, the following reagents were used: normal goat serum (NGS, 10000C, Invitrogen); BLOXALL blocking solution (SP-6000, Vector Laboratories) ; horseradish peroxidase (HRP) conjugated secondary antibody (Vector Laboratories) and 3,3'-Diaminobenzidine (DAB) (SK-4105, Vector Laboratories).

Tamoxifen preparation and induction protocol

Tamoxifen (Sigma) for injection was prepared by first dissolving powder in 100% ethanol. Corn oil was added to make a final concentration of 20mg/ml and ethanol was allowed to completely evaporate under nitrogen stream. Tamoxifen was filter-sterilized and stored at -20°C. For all experiments, 2aEpKO and 2a$^{fl/fl}$ controls were administered with 150µg/g body weight tamoxifen intraperitoneally every other day for a total of 3 injections to delete *Mfsd2a*.

Histological studies using paraffin sections

To prepare skin sections, skin was isolated from the chest (ventral) and back (dorsal) of deeply anaesthetized mice, flattened on a piece of filter paper, and fixed in 4% paraformaldehyde (PFA) in PBS at 4°C overnight. The next day, skin pieces were rinsed in PBS before they were dehydrated in increasing concentrations of ethanol and xylene before embedding in paraffin. Skin sections (5µm) were obtained using microtome (RM2255, Leica) and H&E staining was performed. Images were obtained using BX53 Light Microscope (Olympus). For immunohistochemical analysis, antigen retrieval was performed by boiling sections in sodium citrate buffer, pH6.0 for 5 minutes in a microwave and allowed to cool to room temperature (RT). Skin sections were first blocked with BLOXALL® endogenous blocking solution for 10mins at room temperature followed by blocking buffer (10% NGS, 1% BSA and 0.3M Glycine in TBS-0.1% Triton-X) for 1 hour at room temperature. After blocking, antibodies were diluted in blocking buffer and incubated overnight at 4°C. The following antibodies were used: anti-cytokeratin 5 (1:400), cytokeratin 10 (1:400), Loricrin (1:100) and cytokeratin 6A (1:100). After washing with TBS-Tx, skin sections were incubated with goat anti-rabbit HRP for 1 hour at room temperature and detected using DAB peroxidase substrate following manufacturer's instructions. Reaction was stopped by placing slides immediately in PBS when colour developed, and was not allowed to extend beyond 10mins. Sections were counterstained with hematoxylin and mounted in Permount. Images were obtained using BX53 Light Microscope (Olympus). For immunofluorescence analysis, antigen retrieval was performed by boiling sections in sodium citrate buffer, pH6.0 for 5 minutes in a microwave and allowed to cool to RT. Skin sections were incubated with blocking buffer (10% NGS, 1% BSA and 0.3M Glycine in TBS-0.1% Triton-X) for 1 hour at room temperature. After blocking, antibodies were diluted in blocking buffer and incubated overnight at 4°C. The following antibodies were used: Ki-67 (1:100), and Isolectin GS-IB4 (1:100). After washing with TBS-Tx, tissue sections were incubated with Alexa Fluor secondary antibodies (1:250, ThermoScientific) for 1 hour at RT. Nuclei were stained with Hoechst 33342 (1:500, ThermoFisher Scientific) for 5 mins before mounting with FluorSave™ Reagent (Merck Millipore). Images were obtained using LSM710 Confocal Microscope (Carl Zeiss).

Histological studies using frozen sections

Pregnant mice were euthanized with CO_2 and E18.5 embryos were harvested. Dorsal skin were dissected under stereomicroscope, flattened on a piece of filter paper, and fixed in 4% PFA in PBS at 4°C overnight. The next day, skin pieces were rinsed in PBS before they were cryoprotected in 30% sucrose in PBS overnight and subsequently embedded in OCT (Tissue-Tek; Sakura Finetek USA). Skin sections (12µm) were obtained using Leica Cryostat CM1520. To prepare skin sections from adult mice, skin was isolated from the chest (ventral) and back (dorsal) of deeply anaesthetized mice, flattened on a piece of filter paper, and fixed in 4% paraformaldehyde (PFA) in PBS at 4°C overnight. The next day, tissues were rinsed in PBS and cryoprotected in 30% sucrose in PBS

overnight and subsequently embedded in OCT (Tissue-Tek; Sakura Finetek USA). Skin sections (12μm) were obtained using Leica Cryostat CM1520. For immunofluorescence analysis, tissue sections were rinsed in PBS to remove OCT. Thereafter, tissue sections were incubated with blocking buffer (10% Normal Goat Serum, 1% BSA and 0.3M Glycine in TBS-0.1% Triton-X) for 1 hour at room temperature (RT). After blocking, antibodies were diluted in the same blocking buffer and incubated overnight at 4°C. The following antibodies were used: RFP (1:100), cytokeratin 5 (1:200), cytokeratin 10 (1:200), Filaggrin (1:200), Loricrin (1:100), cytokeratin 6A (1:100), and Isolectin GS-IB4 (1:100). After washing with TBS-Tx, tissue sections were incubated with Alexa Fluor secondary antibodies (1:250, ThermoScientific) for 1 hour at RT. Nuclei were stained with Hoechst 33342 (1:500, ThermoFisher Scientific) for 5 mins before mounting with FluorSave™ Reagent (Merck Millipore). Images were obtained using LSM710 Confocal Microscope (Carl Zeiss).

***In vitro* organotypic epidermis culture system**
Epidermal stratified cultures were carried out as described by Ikuta *et. al.* (2) with modifications. Briefly, a collagen bed containing primary 2a$^{fl/fl}$ mouse dermal fibroblasts were prepared at a density of 1.5 x 10^5 cells/ml. Keratinocytes were isolated from postnatal day 1 2a$^{fl/fl}$ or 2aEpKO mice and 0.4 x 10^5 cells were overlayed onto the collagen bed in hanging well inserts and cultured for 3 days. On day 4, keratinocytes were either treated with 1μM 4-hydroxytamoxifen (4-OHT) or ethanol. The next day, the hanging well insert containing the keratinocytes on the collagen bed was lifted to the air-liquid interface to induce epidermal differentiation. Keratinocytes were allowed to stratify into sheets for 14 days before harvesting for histological analysis. Epidermal sheets and collagen beds were fixed in 4% PFA in PBS at 4°C overnight and processed as before.

Isolation of epidermal sheets for lipidomics analysis and RNA-sequencing
To prepare epidermis for lipidomics analysis, backs of mice were shaved and depilated using hair removal cream (Veet). Dorsal skin explants were harvested from day 13 post-tamoxifen induced 2a$^{fl/fl}$ or 2aEpKO mice or 3 month old WT or 2aKO mice as described by Poumay *et. al.* with modifications (3). After scraping off the underlying subcutaneous fat, skin explants were cut into thin 5mm strips and allowed to float epidermis side up on 10U/ml Dispase II (Gibco) in calcium- and magnesium-free HBSS at 37°C for 1 to 1.5 hours. Epidermis was carefully separated from the dermis using fine-tipped forceps, rinsed briefly in cold calcium- and magnesium-free DPBS and weighed, before carrying out a short spin to remove DPBS. To prepare epidermis for lipidomic analysis, epidermal sheets were flash-frozen in liquid nitrogen and stored at -80°C. To prepare epidermis for RNA-seq, epidermal sheets were harvested similarly as before, but following a quick rinse in in cold calcium- and magnesium-free DPBS, tissues were stored in RNAlater at 4°C overnight. The next day, epidermis was transferred into TRIzol (ThermoFisher Scientific) and stored at -80°C.

Lipidomic analysis and data acquisition (epidermis)
Mass spectrometry-based lipid analysis was performed by Lipotype GmbH (Dresden, Germany) as described (4). Lipids were extracted using a two-step chloroform/methanol procedure (5). Samples were spiked with internal lipid standard mixture containing: cardiolipin 16:1/15:0/15:0/15:0 (CL), diacylglycerol 17:0/17:0 (DAG), hexosylceramide 18:1;2/12:0 (HexCer), lysophosphatidate 17:0 (LPA), lyso-phosphatidylcholine 12:0 (LPC), lysophosphatidylethanolamine 17:1 (LPE), lyso-phosphatidylglycerol 17:1 (LPG), lysophosphatidylinositol 17:1 (LPI), lyso-phosphatidylserine 17:1 (LPS), phosphatidate 17:0/17:0 (PA), phosphatidylcholine 17:0/17:0 (PC), phosphatidylethanolamine 17:0/17:0 (PE), phosphatidylglycerol 17:0/17:0 (PG), phosphatidylinositol 16:0/16:0 (PI), phosphatidylserine 17:0/17:0 (PS), cholesterol ester 20:0 (CE), sphingomyelin 18:1;2/12:0;0 (SM), triacylglycerol 17:0/17:0/17:0 (TAG), omegahydroxy-sphingosine 18:1;2/32:0;0/18:2;0 (EOS D9), omegahydroxyphyto-sphingosine 18:0;3/30:0;0/18:2;0 (EOP), nonhydroxy-sphingosine 18:1;2/18:0;0 (NS D3). After extraction, the organic phase was transferred to an infusion plate and dried in a speed vacuum concentrator. 1st step dry extract was re-suspended in 7.5 mM ammonium acetate in chloroform/methanol/propanol (1:2:4, V:V:V) and 2nd step dry extract in 33% ethanol solution of methylamine in chloroform/methanol (0.003:5:1; V:V:V). All liquid handling steps were performed using Hamilton Robotics STARlet robotic platform with the Anti Droplet Control feature for organic solvents pipetting. Samples were analyzed by direct infusion on a QExactive mass spectrometer (ThermoScientific) equipped with a TriVersa NanoMate ion source (Advion Biosciences). Samples were analyzed in both positive and negative ion modes with a resolution of $R_{m/z=200}$=280000 for MS and $R_{m/z=200}$=17500 for MSMS experiments, in a single acquisition. MSMS was triggered by an inclusion list encompassing corresponding MS mass ranges scanned in 1 Da increments (6). Both MS and MSMS data were combined to monitor CE, DAG and TAG ions as

ammonium adducts; PC, PC O-, Ceramides as acetate adducts; and CL, PA, PE, PE O-, PG, PI and PS as deprotonated anions. MS only was used to monitor LPA, LPE, LPE O-, LPI and LPS as deprotonated anions; HexCer, SM, LPC and LPC O- as acetate adducts. Data were analyzed with Lipotype's in-house developed lipid identification software based on LipidXplorer (7, 8). Data post-processing and normalization were performed using Lipotype's in-house developed data management system. Only lipid identifications with a signal-to-noise ratio >5, and a signal intensity 5-fold higher than in corresponding blank samples were considered for further data analysis. Lipid species were normalized to mol% of their respective classes (sphingolipids, phospholipids or neutral lipids) and used for downstream analysis. Data available in *SI Appendix, Dataset S1*.

Lipidomic analysis (plasma)
Sample preparation for Lipidomics: 10 µL of plasma were combined with 190 µL of butanol:methanol (1:1, v/v) previously spiked with internal standards (ISTD). The standards were purchased from Avanti Lipids and included acylcarnitine 16:0 D3, cholesterol-D7, cholesterol ester 18:0 D6, dihydroceramide d18:0/08:0, ceramide d18:1/12:0, deoxyceramide m18:1/12:0, diacylglycerol 15:0/15:0, GM3 d18:1/18:0 D3, monohexosylceramide d18:1/12:0, dihexosylceramide d18:1/12:0, trihexosylceramide d18:1/18:0 D3, lysophosphatidylcholine 13:0, lysophosphatidylethanolamine 14:0, phosphatidylcholine 13:0/13:0, phosphatidylethanolamine 17:0/17:0, phosphatidylglycerol 17:0/17:0, phosphatidylinositol 12:0/13:0, phosphatidylserine 17:0/17:0, sphingomyelin d18:1/12:0, sphingosine d17:1 and triacylglycerol 12:0/12:0/12:0.
The sample was then vortexed for 10 seconds, sonicated for 30 min and then centrifuged at 14,000 g for 10 min at 4°C. The supernatant was collected for LC-MS/MS analysis. A pooled extract was used as a quality control (QC) sample and injected every five study samples. Data were normalized to original volume of each plasma sample.
*LC-MS/MS analysis:*The LC-MS/MS analysis was performed on an Agilent UHPLC 1290 Infinity II liquid chromatography system connected to an Agilent QqQ 6495C. An Agilent Zorbax RRHD Eclipse Plus C18 column (2.1 × 50 mm, 1.8 µm) was used for the RPLC separation. The mobile phases A (60% water and 40% acetonitrile with 10 mmol/L ammonium formate) and B (10% acetonitrile and 90% isopropanol with 10 mmol/L ammonium formate) were used for the chromatographic analysis. The following gradient was applied: 0-2 min, 20-60% B; 2-12 min, 60-100% B; 12-14 min, 100% B; 14.01-15.8 min, 20% B to equilibrate the column. The oven temperature was maintained at 40°C. Flow rate was set at 0.4 mL/min and the sample injection volume was 2 µL. The positive ionization spray voltage and nozzle voltage were set at 3,000 V and 1,000 V, respectively. The drying gas and sheath gas temperatures were both maintained at 250°C. The drying gas and sheath gas flow rates were 14 L/min and 11 L/min, respectively. The nebulizer nitrogen gas flow rate was set at 35 psi. The iFunnel high and low pressure RF were 150 V and 60 V, respectively.
*Lipidomic Data Analysis:*The acquired MS data were analyzed using Agilent MassHunter software version 10.1. For each analyte, signal to noise ratios (S/N) were calculated using the raw peak areas corresponding to the analytes in QC samples and processed blanks. Lipids associated with S/N < 10, CV > 20% in the QC samples and did not show a linear behaviour (R^2 < 0.8) in dilution curves were excluded from further analysis. Internal standards were used to normalize the raw peak areas for the corresponding lipid class (one internal standard per class) and relative concentrations were further normalized to the protein concentration in the original sample.

RNA-sequencing
To isolate RNA from epidermis, tissues were first lysed in TRIzol (ThermoFisher Scientific) using a MagNA Lyser Instrument (Roche). Total RNA was extracted as described (1). RNA concentration was quantified using Nanodrop. Library preparation and RNA-seq performed by NovogeneAIT (Singapore). 1µg RNA per sample was used for library preparation using NEBNext® Ultra TM RNA Library Prep Kit for Illumina® (NEB, USA) according to manufacturer's instructions, sequenced on the Illumina HiSeq2000 platform and analysis was performed using Partek Flow (version 9). Paired-end reads were aligned to mm10 genome using STAR alignment 2.7.3a and annotated using RefSeq. Features were filtered using recommended parameters and median ratio normalized. Gene expression analysis was performed using DESeq2. Filtered gene lists (genes with fold change ≥ 2, p<0.05 for both 2aKO and 2aEpKO versus their respective controls) were used to perform enrichment analysis on gene sets using Gene Ontology (GO) to identify significantly altered functional groups. DESeq2 normalized counts available in *SI Appendix, Dataset S1*.

Quantitative RT-PCR (qRT-PCR)

RNA was isolated as before and cDNA was synthesized from 1 µg of total RNA using iScript Reverse Transcriptase Supermix (Bio-Rad) according to the manufacturer's recommended protocol. qRT-PCR was performed with the SensiFAST SYBR Hi-ROX Kit (Bioline) using the QuantStudio6Pro (Applied Biosystems). Primer information provided in Table S2. Data was normalized to housekeeping gene ß-actin.

Transmission electron microscopy

2aEpKO and 2a$^{fl/fl}$ mice were anaesthetized, and dorsal skin was isolated. After scraping off the underlying subcutaneous fat, the tissues were orientated and flattened on a piece of filter paper. Skin explants were fixed in 2.5% glutaraldehyde in 0.1 M cacodylate buffer, pH 7.3 at room temperature for 2 hours before trimming and transferring to fresh fixative and allowed to fix at 4°C overnight. Following fixation, skin samples were rinsed 3 times in 0.1 M cacodylate buffer, pH 7.3 and post-fixed in 1% osmium tetroxide, 1.5% potassium ferrocyanide and 3% sucrose for 1 hour at 4°C in the dark. Samples were rinsed 3 times in deionized water and dehydrated in graded series of ethanol (25%, 50%, 75%, 95% and 100%) followed by 100% acetone at room temperature and embedded in 100% Araldite resin (Electron Microscopy Services) at 60°C. Ultra-thin sections of 90 to 100 nm were collected on formvar coated copper grids and stained with 3% uranyl acetate and lead citrate. Samples were viewed with Technai Spirit (FEI company) at 100kV.

Single-cell RNA-sequencing analysis

Publicly available single-cell RNA sequencing (scRNA-seq) datasets were analyzed to assess MFSD2A expression under healthy and diseased conditions. The skin atlas from Reynolds et al. (9) was downloaded in h5ad format from *CellxGene* and imported into R as a Seurat object using the h5ad2seurat function from the schard library. Data processing was performed in *Seurat* (v5.1.4) following *SCTransform* (v0.4.1) normalization. PCA was computed using 100 components, with the top 50 used for UMAP embedding, nearest-neighbor graph construction, and clustering at a resolution of 0.5. Keratinocyte clusters were identified by the expression of *KRT1*, *KRT5*, *KRT10*, and *KRT14*, and subset to evaluate expression of MFSD2A across healthy, psoriasis and eczema samples. The dataset from Ma et al. (10) (GEO: GSE173706) was processed similarly. Ensembl identifiers were converted to HGNC gene symbols using biomaRt. After SCTransform normalization, the top 30 PCs were used for UMAP generation and clustering (resolution = 0.5). Keratinocytes were identified and subset for comparative analysis of MFSD2A expression between healthy, perilesional and lesional psoriasis samples.

Correlation plot

To compare transcriptome perturbations between the 2aEpKO and 2aKO contrasts, fold changes were combined with statistical significance to produce a single rank metric per gene, which was then correlated across contrasts. For each differential expression result, a compound score was computed as: $Compound\ Score = \log_2(Fold\ Change) \times \log_{10}(FDR\ p - value),$ yielding an ordering from most significantly upregulated to most significantly downregulated while jointly weighting effect size and multiple-testing–adjusted significance. This score ranked genes in each comparison, and the two ranked vectors were evaluated for concordance to assess whether genes shift in similar directions across the conditions. The magnitude and direction of association between ranked gene scores from 2aEpKO and 2aKO were quantified using the Spearman rank correlation coefficient, which captures monotonic agreement without assuming linearity or normality of the underlying values.

Table S1. Primers for qRT-PCR (human keratinocytes)

Gene	Forward	Reverse
KRT10	5'-ATTTCTGAGCTGAATCGTGTGATC-3'	5'-CTGATGGACTGCTGCAAGTT-3'
KRT5	5'-CAACCCACTAGTGCCTGGTT-3'	5'-GACACACTTGACTGGCGAGA-3'
KRT10	5'-ATGAGCTGACCCTGACCAAG-3'	5'-TCACATCACCAGTGGACACA-3'
IVL	5'-TCCTCCAGTCAATACCCATCAG-3'	5'-CAGCAGTCATGTGCTTTTCCT-3'
MFSD2A	5'-GTTTCCAGGACCTCAATAGCTC-3'	5'-CAGCAGGTATGCCTTTTGCG-3'
TGM1	5'-GCACCACACAGACGAGTATGA-3'	5'-GGTGATGCGATCAGAGGATTC-3'
FLG	5'-GGACAGGAACAATCATCGGGG-3'	5'-CAACCTCTCGGAGTCGTCTG-3'
RPL13A	5'-CTCAAGGTCGTGCGTCTGAA-3'	5'-TGGCTGTCACTGCCTGGTACT-3'

Table S2. Primers for qRT-PCR (mouse epidermis)

Gene	Forward	Reverse
Mfsd2a	5’-TCTGGTGGGCTTCTGCATTAG-3’	5’-GATGGGAAGTCAGGCACAAAC-3’
ß-actin	5’-GGCTGTATTCCCCTCCATCG-3’	5’-CCAGTTGGTAACAATGCCATGT-3’

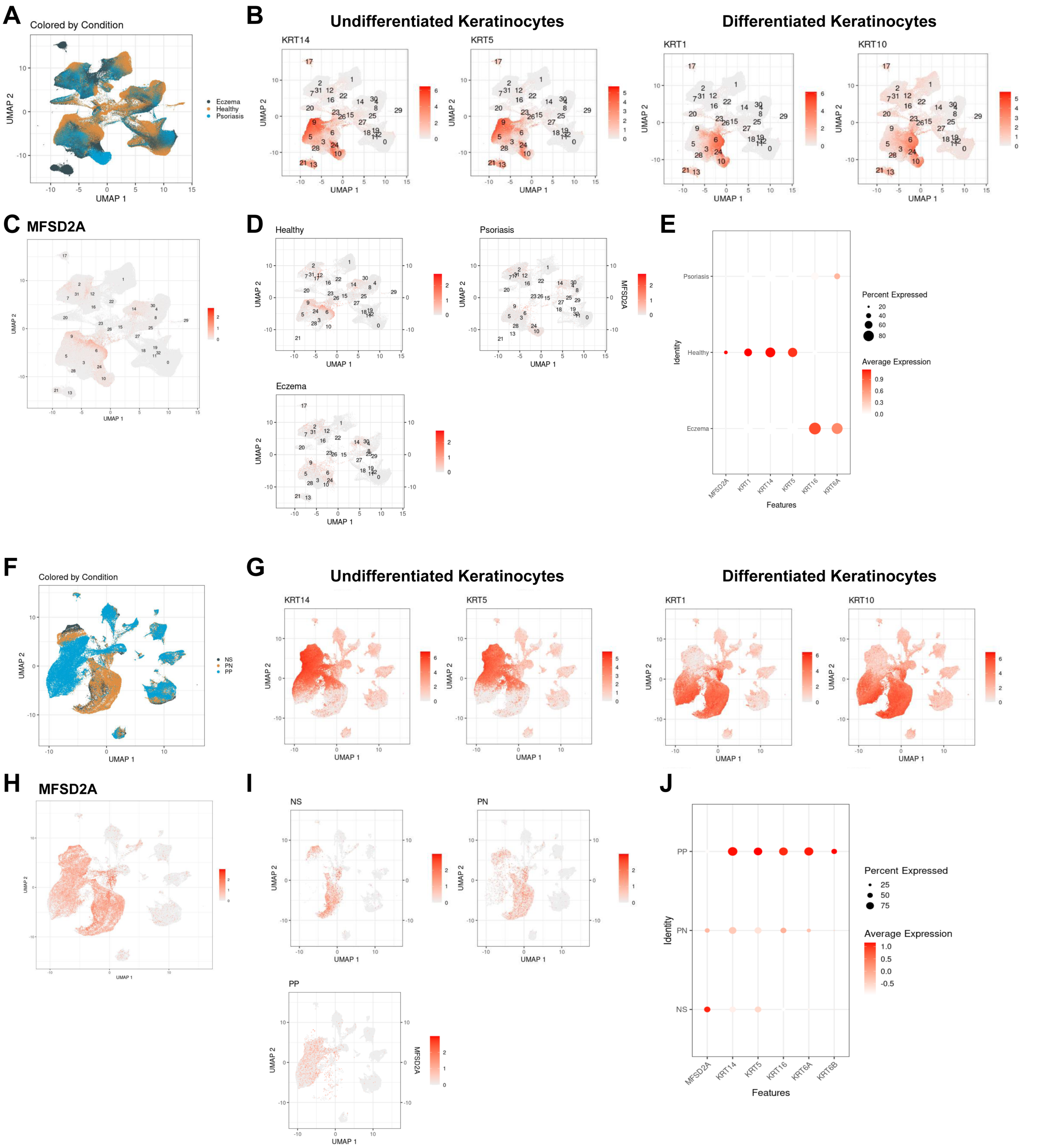

Figure S1. *MFSD2A* is expressed in human keratinocytes and decreased in eczema and psoriasis. Publicly available datasets from Reynolds et. al. (9) **(A-E)** and Ma. et. al. (10) **(F-I)** and were analyzed and represented as UMAP. **(A)** Different cell types in skin colored by disease state. **(B)** Undifferentiated keratinocytes were identified by skin markers Keratin-5 (Krt5) and Keratin-14 (Krt14), while differentiated keratinocytes were identified by skin markers Keratin-1 (Krt1) and Keratin-10 (Krt10). **(C)** *MFSD2A* is expressed primarily in differentiated keratinocytes. *MFSD2A* expression in keratinocytes of healthy, eczema and psoriasis skin represented as UMAP **(D)** and dotplot **(E)**. **(F)** Different cell types in skin colored by disease state. **(G)** Undifferentiated keratinocytes were identified by skin markers Krt5 and Krt14, while differentiated keratinocytes were identified by skin markers Krt1 and Krt10. **(H)** *MFSD2A* is expressed primarily in differentiated keratinocytes. *MFSD2A* expression in keratinocytes of healthy skin (NS), peri-lesional (PN) and lesional (PP) skin represented as UMAP **(I)** and dotplot **(J)**.

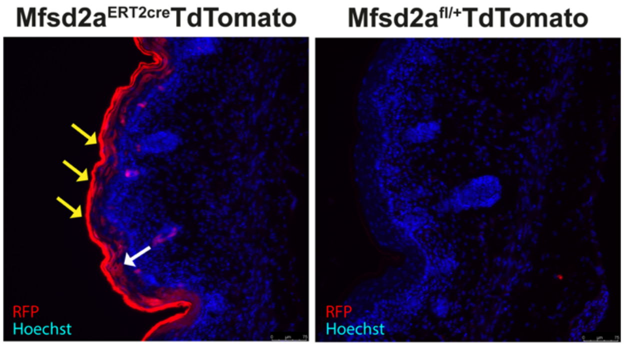

Figure S2. Mfsd2a^{ERT2cre}TdTomato reports *Mfsd2a* expression in the developing epidermis. Female mice at pregnancy day 12 were induced with 3mg Tamoxifen for 3 consecutive days and dorsal skin from E18.5 pups were harvested. Mfsd2a (TdTomato, red) is expressed primarily in the suprabasal (white arrow) and differentiated (yellow arrows) keratinocytes. Mfsd2a$^{fl/+}$TdTomato mice do not express ERT2cre and serves as a negative control. Scale bar = 75μm.

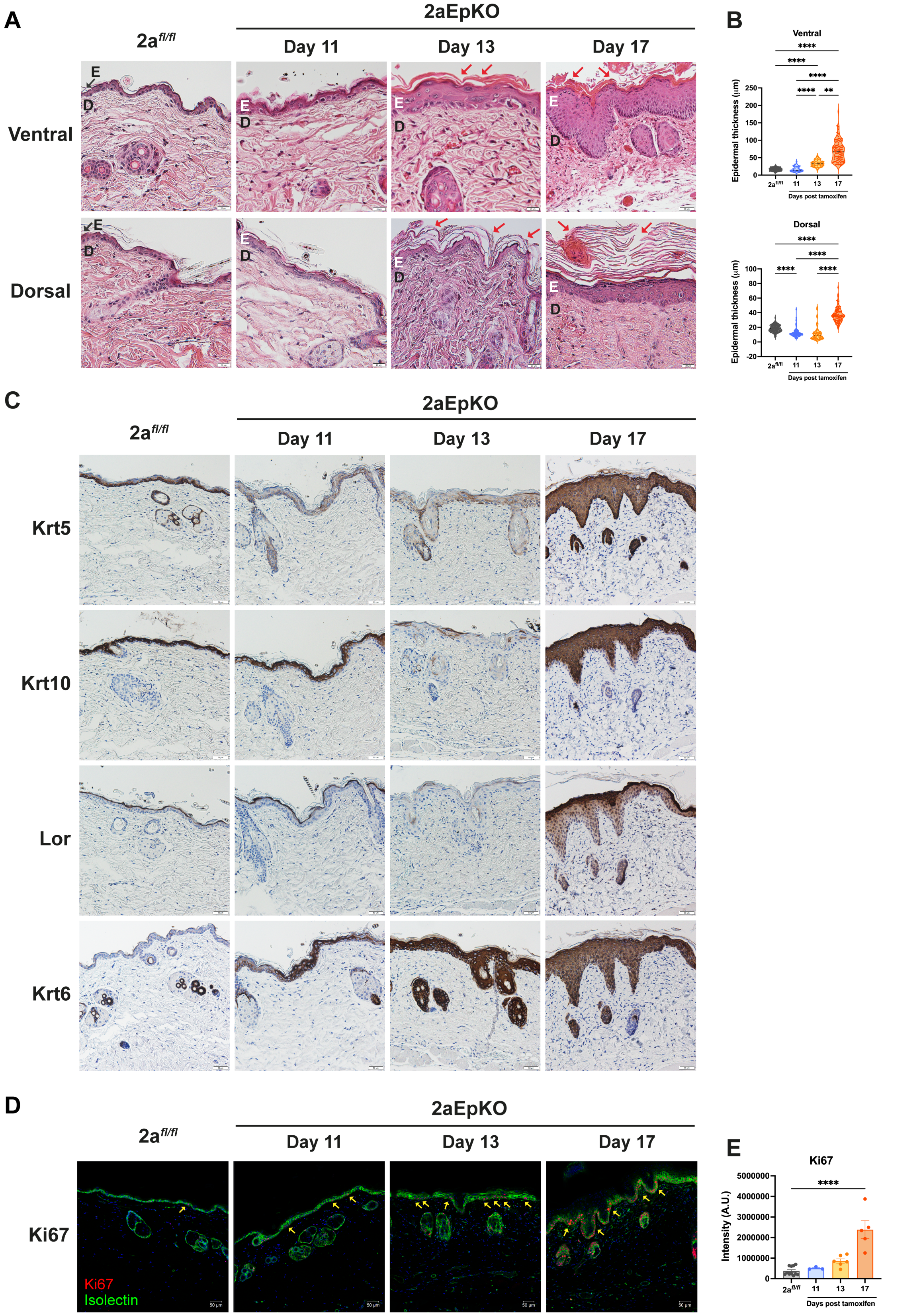

Figure S3. Histological analysis of skin morphology over time following inducible deletion of *Mfsd2a* in 2aEpKO mice. (A) Representative H&E staining of ventral and dorsal skin of 2aEpKO and 2a$^{fl/fl}$ control mice. "Day" indicates number of days post tamoxifen treatment. At day 11 post tamoxifen treatment, 2aEpKO epidermis appears comparable to controls. By day 13, ventral epidermis appears hyperplasic with thick sheets of dead corneocytes (red arrows), while the dorsal

epidermis appeared thin with loosely attached cornified sheets (red arrows). By day 17, 2aEpKO mice developed hyperplasia and hyperkeratosis (red arrows) in both skin depots. **(B)** Violin plot showing epidermal thickness measurements in ventral and dorsal regions of $2a^{fl/fl}$ controls and 2aEpKO. One-way ANOVA with Kruskal-Wallis multiple comparison test. **, p<0.01; ****, p<0.0001. For ventral epidermis quantification, $2a^{fl/fl}$, *n*=10; 2aEpKO day 11, *n*=2; 2aEpKO day 13, *n*=4; 2aEpKO day 17, *n*=4. For dorsal epidermis quantification, $2a^{fl/fl}$, *n*=12; 2aEpKO day 11, *n*=6; 2aEpKO day 13, *n*=7; 2aEpKO day 17, *n*=4. Four measurements were made per field, 3-4 fields were quantified per biological replicate. **(C)** Immunohistochemical staining of epidermal differentiation markers on ventral skin sections. Krt5 (basal keratinocytes), Krt10 (suprabasal keratinocytes) and Loricrin (Lor, terminally differentiated corneocytes). Krt6 (marker for activated keratinocytes). **(D)** Immunohistochemical staining of cell proliferation marker Ki67 (red) on ventral skin sections. Ki67 positive keratinocytes indicated by yellow arrows. Isolectin (green) marks basal cell layer and Hoechst (blue) is a nuclei stain. **(E)** Violin plot showing quantification of Ki67 expression in $2a^{fl/fl}$ and 2aEpKO epidermis. One-way ANOVA with Dunnett's multiple comparison test. ****, p<0.0001. $2a^{fl/fl}$, *n*=4; 2aEpKO day 11, *n*=2; 2aEpKO day 13, *n*=2; 2aEpKO day 17, *n*=2. 2-3 fields were quantified per biological replicate. Scale bar = 20μm, 50μm.

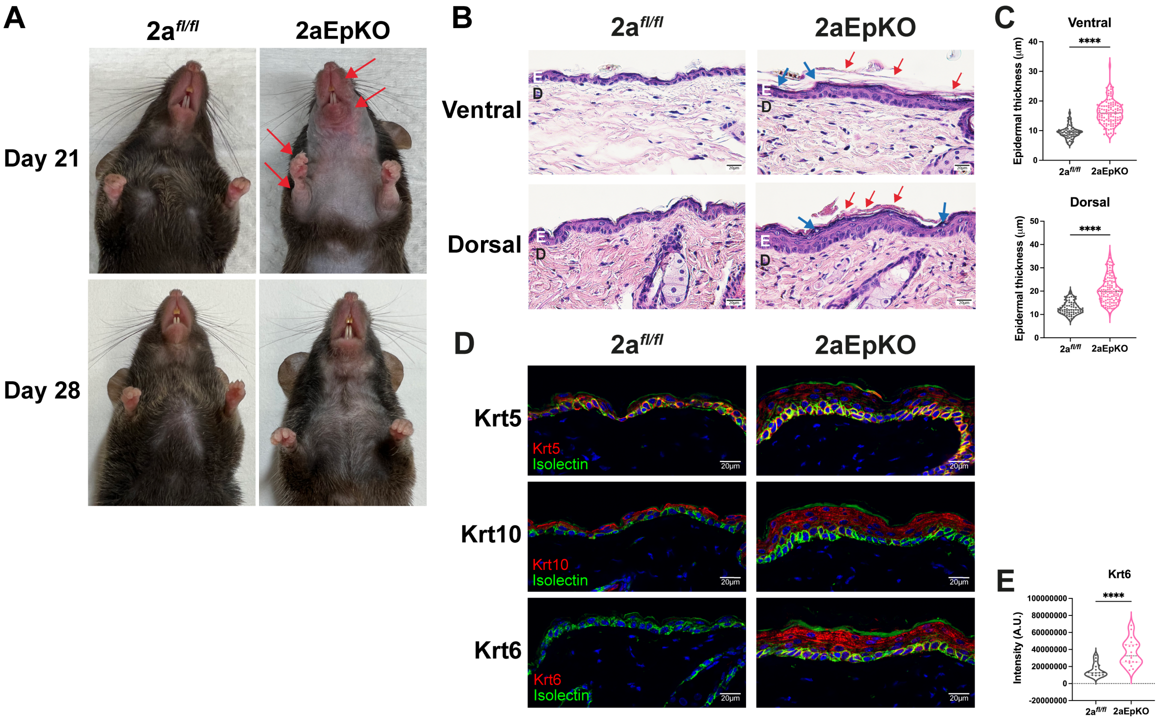

Figure S4. Dermatitis but not epidermal desquamation defects resolved by day 28 post tamoxifen treatment in 2aEpKO mice. (A) Photos of mice at day 21 and 28 post tamoxifen treatment. 2aEpKO appeared mildly dermatotic at areas indicated by red arrows, while 2a$^{fl/fl}$ controls appeared normal. By day 28 post tamoxifen treatment, alopecia and dermatotic phenotypes in 2aEpKO mice resolved. (**B**) Representative image of H&E stained ventral and dorsal skin of 2a$^{fl/fl}$ control and 2aEpKO mice. 2aEpKO mice still appeared hyperplasic and hyperkeratotic (red arrows) with parakeratosis (blue arrow). **(C)** Violin plot showing ventral and dorsal epidermal thickness measurements of 2a$^{fl/fl}$ and 2aEpKO. Four measurements were made per field, 3-6 fields were quantified per biological replicate. Unpaired T-test with Welch's correction, ****, $p<0.0001$. **(D)** Immunohistochemical staining of indicated epidermal differentiation markers. Krt5 (basal keratinocytes), Krt10 (suprabasal keratinocytes), Krt6 (marker for activated keratinocytes). Isolectin (green) marks basal layer and Hoechst (blue) is a nuclei stain. E, epidermis; D, dermis. Scale bar = 20μm. **(E)** Violin plot showing quantification of Krt6 expression in dorsal epidermis of 2a$^{fl/fl}$ controls and 2aEpKO. Unpaired T-test with Welch's correction, ****, $p<0.0001$. For all panels, *n*=5 per genotype.

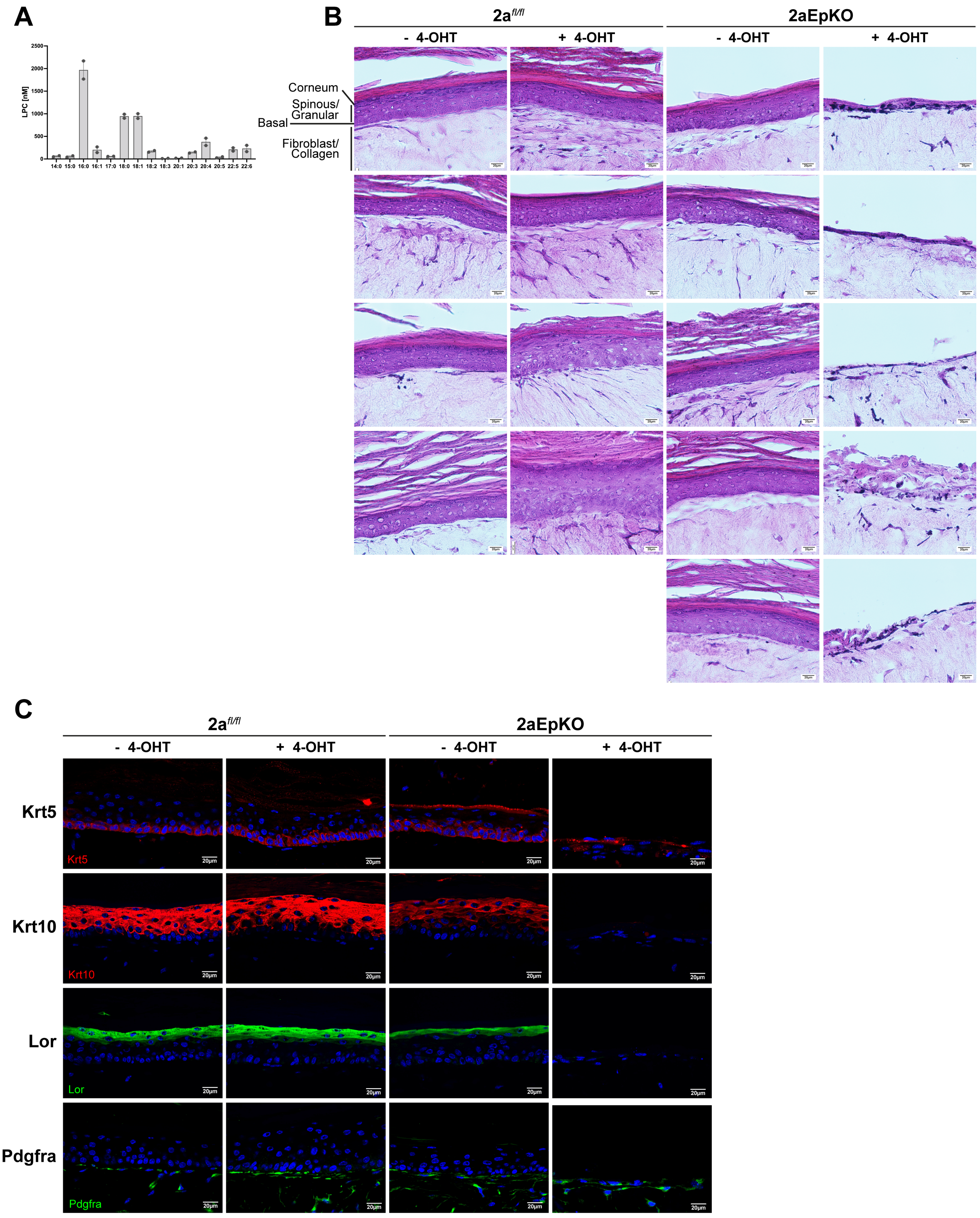

Figure S5. Mfsd2a is essential for epidermal stratification *in vitro*. (A) LPC concentration in media used to stratify keratinocytes; data are represented as scatterplot with bars. n=2, technical replicates. **(B)** Primary keratinocytes isolated from the same mouse (2a$^{fl/fl}$ and 2aEpKO) was split into two group, treated and untreated with 4-hydroxytamoxifen (4-OHT). Representative images of H&E stained stratified epidermal sheets after 14 days of air exposure. 2a$^{fl/fl}$, *n*=4 mice; 2aEpKO, *n*=5 mice. Scale bar = 20µm. **(C)** Immunohistochemical staining of indicated epidermal differentiation markers. Krt5 (basal keratinocytes), Krt10 (suprabasal keratinocytes) and Loricrin (Lor, terminally differentiated corneocytes). Pdgfra, a fibroblast marker to denote the dermis. Hoechst (blue) is a nuclei stain; 2a$^{fl/fl}$, *n*=4 mice; 2aEpKO, *n*=5 mice. Scale bar = 20µm.

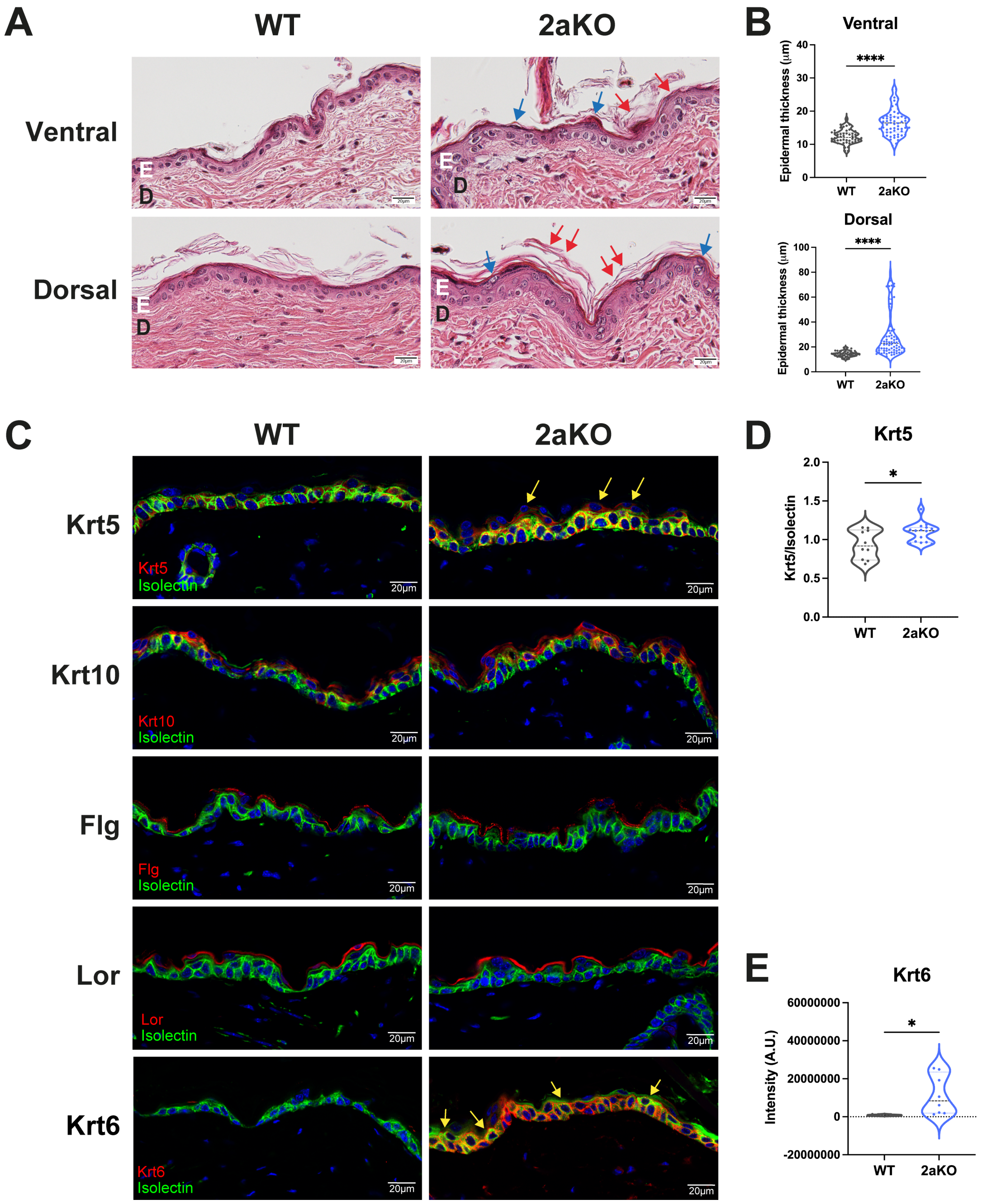

Figure S6. Mice with conventional deficiency of Mfsd2a have a defect in epidermal desquamation. (A) Representative H&E staining of ventral and dorsal skin of 3 month old WT and 2aKO mice. Mild hyperkeratosis observed in 2aKO epidermis relative to WT. Thick sheets of dead corneocytes (red arrows) and parakeratosis (blue arrows) in stratum corneum layer indicate desquamation defects in 2aKO. **(B)** Violin plot showing epidermal thickness measurements of ventral and dorsal skin of WT and 2aKO mice. Four measurements were made per field, 3-4 fields were quantified per biological replicate. Unpaired T-test with Welch's correction, ****, p<0.0001. **(C)** Immunohistochemical staining of epidermal differentiation markers (red) on dorsal skin sections. Krt5 (basal keratinocytes), Krt10 (suprabasal keratinocytes), Filaggrin (Flg, granular keratinocytes) and Loricrin (Lor, terminally differentiated corneocytes). Krt6 (marker for activated keratinocytes). Isolectin (green) marks basal cell layer and Hoechst (blue) is a nuclei stain. Suprabasal cells of 2aKO epidermis retain Krt5 expression (yellow arrows). **(D)** Violin plot showing quantification of Krt5/Isolectin expression in WT and 2aKO epidermis. Unpaired T-test with Welch's correction, *, p<0.05. **(E)** Violin plot showing quantification of Krt6 expression in WT and 2aKO epidermis. Unpaired T-test with Welch's correction, *, p<0.05. For all panels, *n*=4-5 per genotype; biological replicates. Scale bar = 20μm.

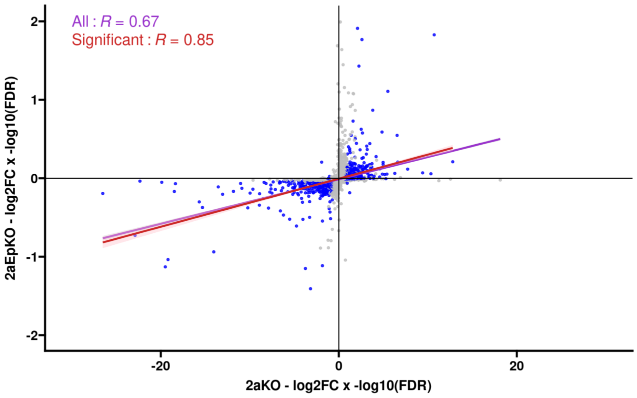

Figure S7. Changes in the epidermal transcriptome of 2aEpKO mirror changes observed in 2aKO. RNA-seq analysis of 2aEpKO and 2aKO epidermis versus their respective controls. Correlation plot of $\log_2$(fold change) x $-\log_{10}$(FDR p-value) of all genes in 2aEpKO (y-axis) versus 2aKO (x-axis) represented as grey dots. Pearson correlation coefficient indicated by purple line. Significantly different genes that are observed in both 2aEpKO and 2aKO are represented as blue dots. Spearman correlation coefficient indicated by red line and a r = 0.85 indicates a strong positive correlation between both genotypes.

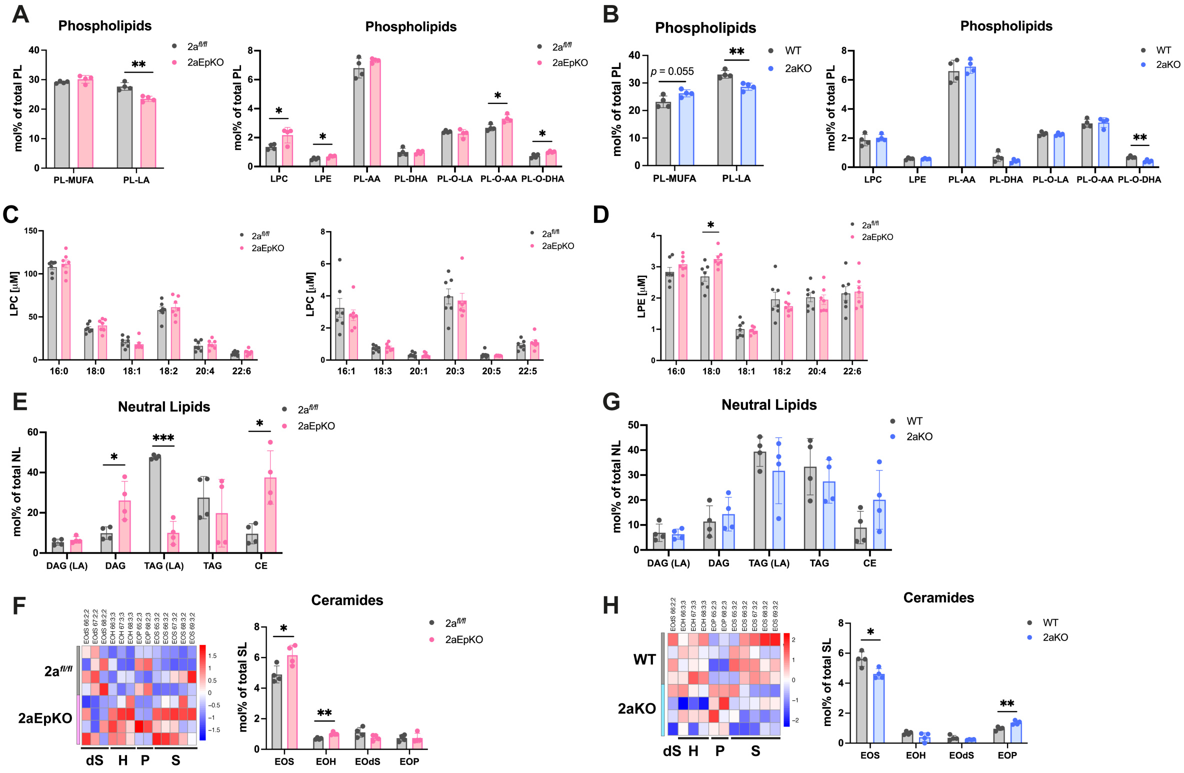

Figure S8. Mfsd2a deficiency alters the epidermal lipidome. Scatterplot with bars showing lipid changes in epidermis of 2aEpKO **(A, E and F)** and 2aKO **(B, G and H)** mice versus their respective controls. Epidermal lipid species represented as mol% of phospholipids (PL), neutral lipids (NL) or sphingolipids (SL). Plasma LPC **(C)** and LPE **(D)** concentrations in 2aEpKO and $2a^{fl/fl}$ controls represented as scatterplot with bars. Common LPC and LPE species are shown with fatty acyl chain indicated. Heatmap representation of log_2-transformed fold change in phospholipids of 2aEpKO **(F)** and 2aKO **(H)** relative to their respective controls. Color bar indicates z-score transformation on mol% SL. For panels A, B, E-H, individual points in bar graphs indicate biological replicates with data represented as mean ± S.E. *n*=4 per genotype. For panels C and D, individual points in bar graphs indicate biological replicates with data represented as mean ± S.E. *n*=7 per genotype. Unpaired T-test with Welch's correction, *, p<0.05; **, p<0.01; ***, p<0.001.

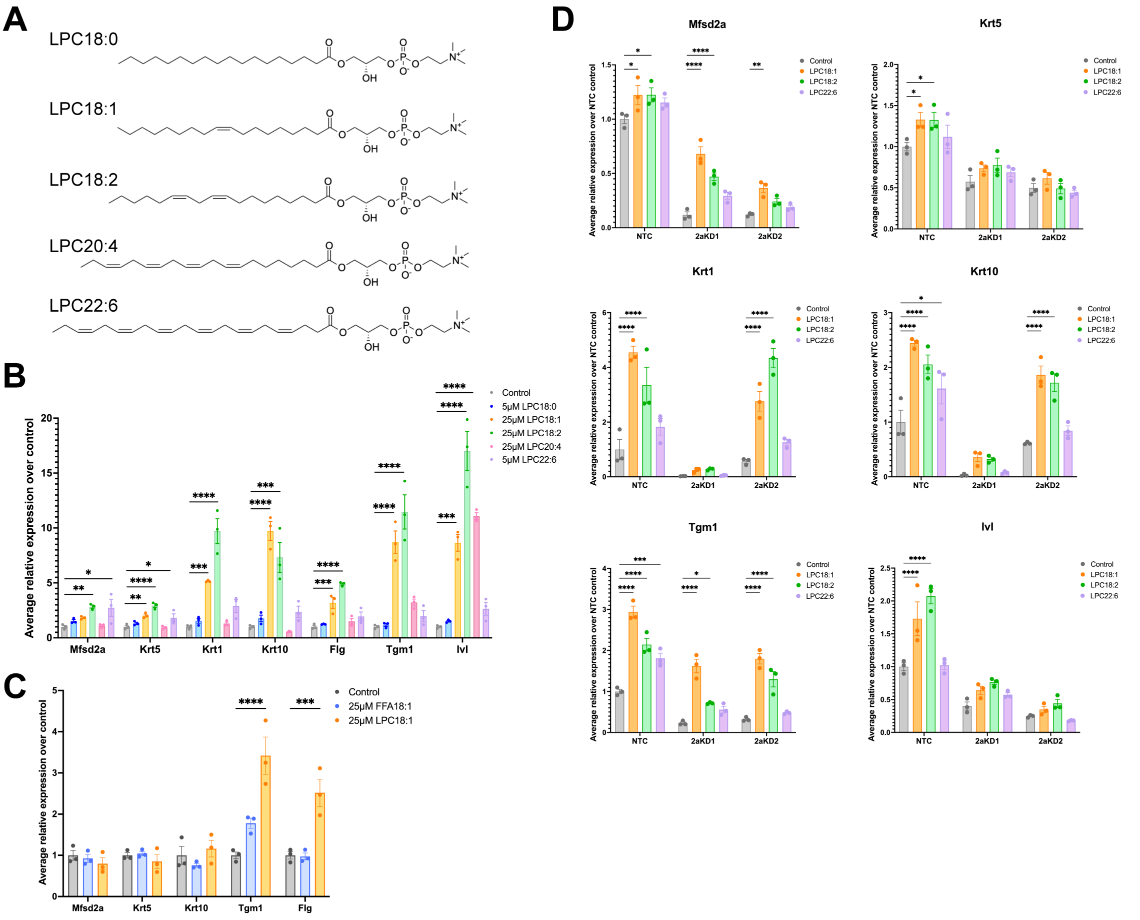

Figure S9. LPC uptake by Mfsd2a enhances primary human keratinocyte differentiation. (A) Lipid structures of LPCs used to treat keratinocytes in Fig. 5A, B, E, F. **(B)** Scatterplot with bars showing fold changes of epidermal differentiation markers in LPC treated versus control keratinocytes. Individual points indicate each technical replicate with data shown as mean ± S.E. *n*=3 per treatment, technical replicates. Two-way ANOVA with Dunnett's multiple comparison test. *, p<0.05; **, p<0.01; ***, p<0.001; ****, p<0.0001. **(C)** Scatterplot with bars showing fold changes of epidermal differentiation markers in keratinocytes treated with either LPC-18:1 or unesterified fatty acid 18:1 (FFA18:1). Individual points indicate each technical replicate with data shown as mean ± S.E. *n*=3 per treatment. Two-way ANOVA with Tukey's multiple comparison test. ***, p<0.001; ****, p<0.0001. **(D)** Scatterplot with bars showing fold changes of epidermal differentiation markers in different treatment group versus NTC control keratinocytes. Individual points indicate each technical replicate with data represented as mean ± S.E. *n*=3 per treatment, technical replicates. Two-way ANOVA with Tukey's multiple comparison test. *, p<0.05; **, p<0.01; ***, p<0.001; ****, p<0.0001.

SI References:

1. J. P. Chan *et al.*, The lysolipid transporter Mfsd2a regulates lipogenesis in the developing brain. *PLoS Biol* **16**, e2006443 (2018).
2. S. Ikuta, N. Sekino, T. Hara, Y. Saito, K. Chida, Mouse epidermal keratinocytes in three-dimensional organotypic coculture with dermal fibroblasts form a stratified sheet resembling skin. *Biosci Biotechnol Biochem* **70**, 2669-2675 (2006).
3. Y. Poumay, I. H. Roland, M. Leclercq-Smekens, R. Leloup, Basal detachment of the epidermis using dispase: tissue spatial organization and fate of integrin alpha 6 beta 4 and hemidesmosomes. *J Invest Dermatol* **102**, 111-117 (1994).
4. J. L. Sampaio *et al.*, Membrane lipidome of an epithelial cell line. *Proc Natl Acad Sci U S A* **108**, 1903-1907 (2011).
5. C. S. Ejsing *et al.*, Global analysis of the yeast lipidome by quantitative shotgun mass spectrometry. *Proc Natl Acad Sci U S A* **106**, 2136-2141 (2009).
6. M. A. Surma *et al.*, An automated shotgun lipidomics platform for high throughput, comprehensive, and quantitative analysis of blood plasma intact lipids. *Eur J Lipid Sci Technol* **117**, 1540-1549 (2015).
7. R. Herzog *et al.*, LipidXplorer: a software for consensual cross-platform lipidomics. *PLoS One* **7**, e29851 (2012).
8. R. Herzog *et al.*, A novel informatics concept for high-throughput shotgun lipidomics based on the molecular fragmentation query language. *Genome Biol* **12**, R8 (2011).
9. G. Reynolds *et al.*, Developmental cell programs are co-opted in inflammatory skin disease. *Science* **371** (2021).
10. F. Ma *et al.*, Single cell and spatial sequencing define processes by which keratinocytes and fibroblasts amplify inflammatory responses in psoriasis. *Nat Commun* **14**, 3455 (2023).
